# The effects of membrane potential oscillations on the excitability of rat hypoglossal motoneurons

**DOI:** 10.3389/fphys.2022.955566

**Published:** 2022-08-23

**Authors:** Qiang Zhang, Yue Dai, Junya Zhou, Renkai Ge, Yiyun Hua, Randall K. Powers, Marc D. Binder

**Affiliations:** ^1^ Shanghai Key Laboratory of Multidimensional Information Processing, School of Communication and Electronic Engineering, East China Normal University, Shanghai, China; ^2^ Key Laboratory of Adolescent Health Assessment and Exercise Intervention of Ministry of Education, School of Physical Education and Health Care, East China Normal University, Shanghai, China; ^3^ School of Physical Education and Health Care, East China Jiaotong University, Nanchang, China; ^4^ Neuroscience, McGill University, Montreal, QC, Canada; ^5^ Department of Physiology & Biophysics, School of Medicine, University of Washington, Seattle, WA, United States

**Keywords:** hypoglossal motoneurons, motor control, membrane oscillations, excitability, modeling

## Abstract

Oscillations in membrane potential induced by synaptic inputs and intrinsic ion channel activity play a role in regulating neuronal excitability, but the precise mechanisms underlying their contributions remain largely unknown. Here we used electrophysiological and modeling approaches to investigate the effects of Gaussian white noise injected currents on the membrane properties and discharge characteristics of hypoglossal (HG) motoneurons in P16-21 day old rats. We found that the noise-induced membrane potential oscillations facilitated spike initiation by hyperpolarizing the cells’ voltage threshold by 3.1 ± 1.0 mV and reducing the recruitment current for the tonic discharges by 0.26 ± 0.1 nA, on average (*n* = 59). Further analysis revealed that the noise reduced both recruitment and decruitment currents by 0.26 ± 0.13 and 0.33 ± 0.1 nA, respectively, and prolonged the repetitive firing. The noise also increased the slopes of frequency-current (F-I) relationships by 1.1 ± 0.2 Hz/nA. To investigate the potential mechanisms underlying these findings, we constructed a series of HG motoneuron models based on their electrophysiological properties. The models consisted of five compartments endowed with transient sodium (NaT), delayed-rectify potassium [K(DR)], persistent sodium (NaP), calcium-activated potassium [K(AHP)], L-type calcium (CaL) and H-current channels. In general, all our experimental results could be well fitted by the models, however, a modification of standard Hodgkin-Huxley kinetics was required to reproduce the changes in the F-I relationships and the prolonged discharge firing. This modification, corresponding to the noise generated by the stochastic flicker of voltage-gated ion channels (channel flicker, CF), was an adjustable sinusoidal function added to kinetics of the channels that increased their sensitivity to subthreshold membrane potential oscillations. Models with CF added to NaP and CaL channels mimicked the noise-induced alterations of membrane properties, whereas models with CF added to NaT and K(DR) were particularly effective in reproducing the noise-induced changes for repetitive firing observed in the real motoneurons. Further analysis indicated that the modified channel kinetics enhanced NaP- and CaL-mediated inward currents thus increasing the excitability and output of HG motoneurons, whereas they produced relatively small changes in NaT and K(DR), thus balancing these two currents and triggering variability of repetitive firing. This study provided insight into the types of membrane channel mechanisms that might underlie oscillation-induced alterations of neuronal excitability and motor output in rat HG motoneurons.

## 1 Introduction

Neural transmission mainly depends on the integration of synaptic inputs. The activation of presynaptic neurons normally induces transient changes in the discharge probability of postsynaptic neurons. The presynaptic inputs are composed of excitatory and inhibitory transient currents and generate membrane potential oscillations in postsynaptic neurons that are generated by excitatory postsynaptic potentials (EPSPs) and inhibitory postsynaptic potentials (IPSPs). Membrane potential oscillation is one of primary modulators of spike initiation and neuronal excitability (Poliakov et al., 1996; Powers and Türker, 2010). In spinal motoneurons, membrane oscillations are observed prior to the full establishment of fictive locomotion induced by electrical stimulation of the mesencephalic locomotor region in decerebrate cats (Krawitz et al., 2001) and rats (MacDonell et al., 2015). These oscillations were considered to a facilitatory prelude to motoneuron activation in locomotion. In addition to synaptic inputs, membrane potential oscillations can also be generated by the intrinsic properties of membrane channels (Powers et al., 2008). It has been reported that the generation of subthreshold membrane potential oscillations in rat DRG neurons depends on the TTX-sensitive sodium currents (Amir et al., 1999). The TTX-sensitive persistent sodium currents mediate the subthreshold membrane potential oscillations and rhythmic firings, and such oscillations are inhibited by the addition of low concentrations of TTX (Cummins et al., 1998; Hutcheon and Yarom, 2000; Agrawal et al., 2001). Monoamine-triggered membrane potential oscillations have also been reported in spinal interneurons, and these oscillations are thought to facilitate spike initiation and tonic discharge (Amitai, 1994). In addition, acetylcholine-induced membrane potential oscillations were observed in ascending commissural interneurons in neonatal mice. These oscillations largely increased the firing frequency of the neurons and were shown to be TTX insensitive, suggesting that voltage-gated sodium channels are not required (Carlin et al., 2006; Zhong et al., 2006). Similar observations of serotonin-induced membrane potential oscillations were also reported in mouse spinal cord neurons active during locomotion (Dai et al., 2009). Taken together, these results suggest that several species of membrane channels are implicated in the generation of membrane potential oscillations that affect the excitability and discharge patterns of neurons.

The functional roles of membrane potential oscillations induced by white noise have been studied intensively in rat hypoglossal (HG) motoneurons, focusing on the probability of spike initiation and frequency of repetitive firing (Poliakov et al., 1996; Poliakov et al., 1997; Türker and Powers 1999). These combined experimental and simulation studies provide insight into mechanisms underlying the repetitive firing of HG motoneurons. However, a complete understanding of the regulation of excitability and the input-output properties of HG motoneurons continues to elude us. In particular, the effects of membrane potential fluctuations on the gating behavior of motoneuronal voltage-gated channels remain unexplored.

Previous studies have characterized a host of voltage-gated membrane channels in juvenile rat hypoglossal motoneurons, including voltage-gated sodium, potassium (Lape and Nistri, 1999; Lape and Nistri, 2001; Powers and Binder 2003; Viana et al., 1993a; Viana et al., 1993b), and several different types of calcium channels (Powers and Binder 2003; Umemiya and Berger, 1994; Umemiya and Berger, 1995; Viana et al., 1993a: Viana et al., 1993b; Moritz et al., 2007). In this study, using combined experimental and modeling approaches we have investigated the effect of noise-induced membrane potential oscillations on the excitability and discharge behavior of rat HG motoneurons. Membrane potential oscillations were induced in rat and modeled HG motoneurons by injecting a zero-mean, white noise waveform emulating excitatory and inhibitory transient currents into the soma. The experimental results demonstrated that white noise increased excitability and output of HG motoneurons. In general, all our experimental results could be well fit by the models, however, a modification of standard Hodgkin-Huxley kinetics was required to reproduce the changes in the F-I relationships and the prolonged discharge firing. This modification, corresponding to the noise generated by the stochastic flicker of voltage-gated ion channels (channel flicker, CF, White et al., 2000; Dorval and White, 2005; Dorval, 2006), was an adjustable sinusoidal function added to kinetics of the channels that increased their sensitivity to subthreshold membrane potential oscillations.

## 2 Materials and methods

### 2.1 Experimental recordings

Experiments were carried out in accordance with the animal welfare guidelines at the University of Washington with protocol approval from the IACUC Committee. Sprague-Dawley rats of postnatal day 16–21 were anesthetized by an intramuscular injection of 1.8 ml/kg of a 5:1.6:6.6 solution of ketamine: xylazine: saline. A section of the brainstem from the mid-medulla to the rostral pons was removed, and 400 μm, transverse slices containing the hypoglossal (HG) nucleus were cut, as previously described (Powers et al., 2005). Individual slices were transferred from a holding chamber to the recording chamber submerged in artificial cerebrospinal fluid (ACSF) at room temperature flowing at a rate of 2 ml/min. Intracellular recordings were made in hypoglossal motoneurons (HM) from brainstem slices. The ACSF contained (in mM): 126 NaCl, 2 KCl, 1.25 NaH_2_PO_4_, 26 NaHCO_3_, 2 MgCl_2_, 2 CaCl_2_, and 10 glucose. The HG nucleus was identified visually by anatomic position in the slice, and intracellular recordings were obtained using glass sharp electrodes filled with 3 M KCl or K-acetate, with resistances of 20–50 MΩ. Motoneuron identification was based on location and on the similarity of their intrinsic properties to those previously reported (e.g., Viana et al., 1990).

Intrinsic motoneuron membrane properties were measured including the rheobase obtained by the minimum amplitude of a 50 ms injected current pulse needed to elicit a spike, input resistance, and the amplitude and duration of the afterhyperpolarization (AHP) following single spikes elicited at several different mean membrane potentials. The frequency-current (F-I) relationship was established in response to a series of 1 s current pulses of different amplitude, and voltage threshold (V_th_) of spikes induced by a family of bi-ramp currents (5 s, ramp peak increment 200 pA) was defined as the membrane potential at which the rising rate of dV/dt ≥10 mV/ms. The recruitment current (I_rec_) was defined as the point of depolarizing bi-ramp current at which the first spike was initiated, and the decruitment current (I_dec_) as the point of repolarizing bi-ramp current at which the last spike was generated. We calculated the difference ΔI = I_dec_–I_rec_.

We obtained a series of motoneuronal responses to a noisy injected current waveform (described below) superimposed on a current step of different amplitudes and a family of bi-ramp currents. The command for the injected current waveform was computed and stored as a wave in Igor (Wavemetrics, Oswego, OR) and output *via* an Instrutech D/A converter at a sampling rate of 10 KHz. This output waveform was sent to the external current command of an Axoclamp 2B amplifier, operating in either bridge or discontinuous current clamp mode, and the resultant voltage response was also sampled at a rate of 10 KHz and stored as an Igor wave. Student’s T-tests were performed on our data with statistical significance defined as *p* < 0.05. Results are shown as mean ± SD.

### 2.2 Stimulus waveform

The injected current waveform consisted of the following components: 1) the current steps of 1 s, 2) a family of bi-ramp currents of 6 s with ramp peaks of 0.2, 0.4, 0.6, 0.8, 1.0, and 1.2 nA which correspond to slopes of 0.06, 0.13, 0.20, 0.26, 0.33, and 0.4 nA/s; 3) a zero-mean random noise waveform, 4) the sum of (1) & (3) or (2) & (3), (5) the trains of current transients composed of excitatory and inhibitory inputs starting at the same time as the noise waveform, and (6) two series of eight 1 ms, 1 nA hyperpolarizing current pulses applied before and after the current step. The random noise component was filtered, Gaussian noise generated with the following recursive formula:

$$X\left(i\right)=\left(1-\Delta t/\tau_{f}\right)\times X\left(i-1\right)+gnoise\left(\sigma \right)\times \sqrt{1-{\left(1-\Delta t/\tau_{f}\right)}^{2}}$$ (1)

where *X(i)* is the ith point of the random waveform, *gnoise(σ)* is a random number drawn from a Gaussian distribution with a zero-mean of and a standard deviation of σ, and τ_f_ is the filtering time constant. For all trials, the noise standard deviation was set to 0.25 nA and the filtering time constant was 1 ms (τ_f_ = 10 ms at our sampling rate of 10 KHz), but we varied the exact values of the noise from trial to trial by choosing different random number seeds. The trains of transients were two symmetric (positive and negative) alpha functions applied at a mean rate of 40 Hz, with Poisson distributions of intervals. The time-to-peak of the alpha functions was 0.5 ms and the peak amplitudes were ±0.3 nA. The sum of the transient trains and the random noise component also exhibited a Gaussian amplitude distribution, but with a standard deviation of 0.262 nA.

#### 2.3 Modeling

**FIGURE 1:**
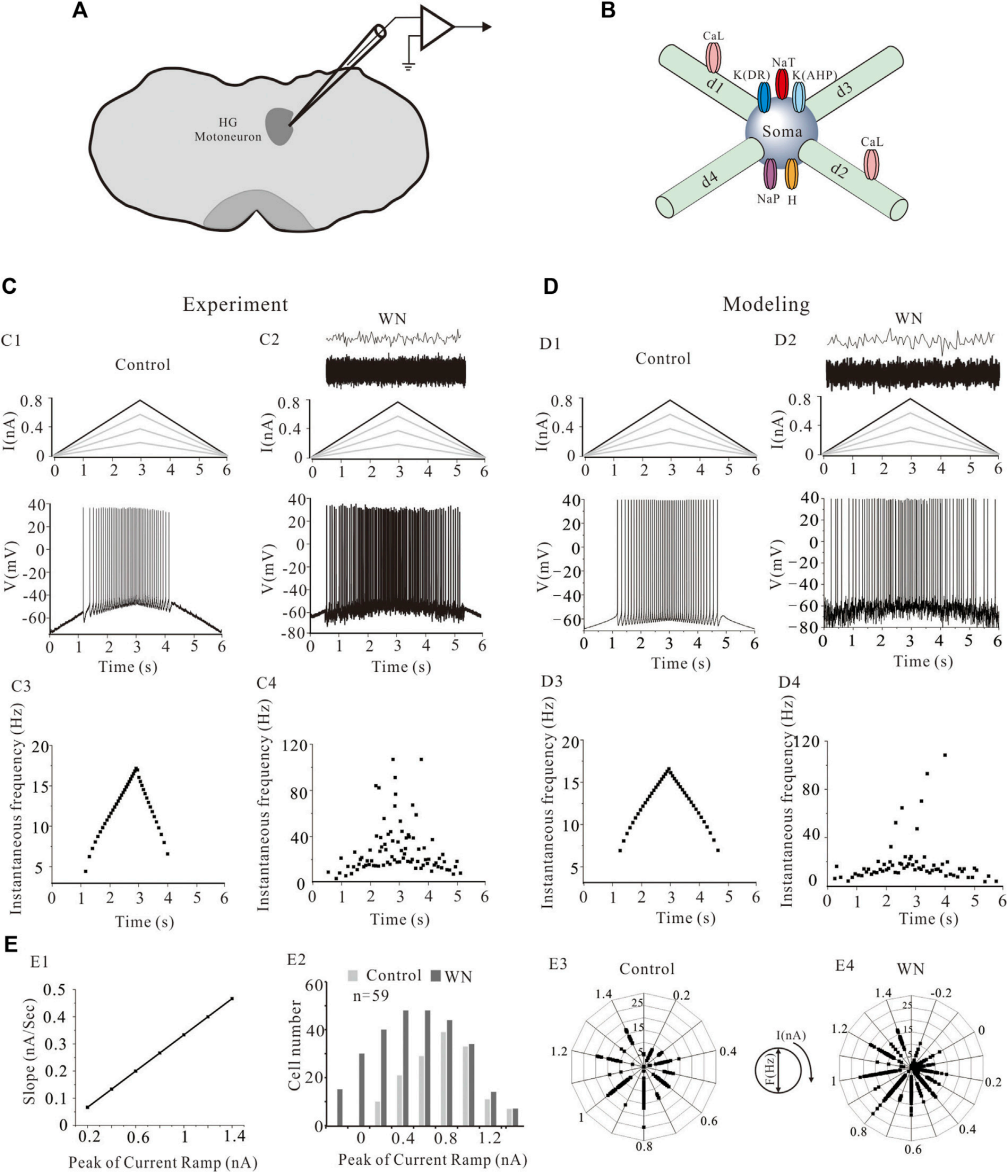
The location of hypoglossal (HG) motoneurons and a simplified HG motoneuron model. **(A)** The preparation of rat brainstem slices and the location of hypoglossal nucleus. **(B)** Five HG motoneuron models were built and each model consisted of soma and four dendritic compartments (d1, d2, d3, and d4). The soma contained transient sodium (NaT), persistent sodium (NaT), delayed-rectifier potassium (K(DR)), Ca^2+^-dependent potassium (K(AHP)) and H-current channels. L-type calcium channels (CaL) were distributed on the d1 and d2 dendritic compartments. **(C)**: A family of bi-ramp currents with duration of 6 s and step of 0.2 nA ramp peak were applied to HG motoneurons and models (**C1**, top). A bi-ramp current with a duration of 6 s, peak of 0.8 nA was injected into HG motorneuron without (**C1**, top) and with white noise (WN) (**C2**, top) and repetitive firings were evoked without (**C1**, bottom) and with (**C2**, bottom) WN. The white noise superimposed by current transients (**C2**, top) was injected into the motoneuron. Instantaneous firing frequencies were calculated without **(C3)** and with WN **(C4)** from corresponding firings **(C1,C2)**, respectively. **(D)**: Similar recordings from simulation protocol. A bi-ramp current with a duration of 6 s, peak of 0.8 nA was injected into a HG motoneuron model without (**D1**, top) and with white noise (**D2**, top) to generate repetitive firing (**D1**, bottom; **D2**, bottom). Instantaneous firing frequencies without **(D3)** and with **(D4)** WN were calculated from corresponding firings **(D1)** and **(D2)**, respectively. **(E)**: The relationship between slopes of rising phase of the ramps and the step of ramp peaks was established **(E1)**. The number of cells recorded at each step of the ramps was calculated from total of 59 cells in both control and white noise **(E2)**. Frequency distribution of the firings at each ramp peak was calculated in control **(E3)** and white noise **(E4)**.

Figure 1A shows a graphic representation of the electrical recording set up. Hypoglossal motoneuron models were built based on the experimental data (Viana et al., 1994) with NEURON 7.7 (Tables 1, 2). In an attempt to reproduce the data we collected from our physiological experiments (Table 1), we constructed five different HG motoneuron models to encompass the dynamic range of motoneuron firing properties that we observed in the sample of HG motoneurons that we studied in rats. In each model, we included voltage-dependent transient sodium (NaT), persistent sodium (NaP), delayed-rectifier potassium K(DR)), Ca^2+^-activated K^+^ (K(AHP)), and hyperpolarization-activated inward currents (H) that have been widely found in mammalian motoneurons, including HG motoneurons, and are major channels responsible for generation of action potentials and repetitive discharges. The rationale for the inclusion of these channels in the present models was based on previous studies on. The properties of these channels in our models matched those reported in the previous studies of rat HG motoneurons including CaL (Viana et al., 1993a; Viana et al., 1994; Moritz et al., 2007), K(DR) (Viana et al., 1993b) and NaP (Zeng et al., 2005). Parameter optimization of the HH-type equations was based on the previous modeling studies (Dai et al., 2002; Powers et al., 2012; Power and Heckman, 2015; Dai et al., 2018), as well as the “target values” of rat HG motoneurons in Table 1. Each model included five-compartments, a somatic compartment and four dendritic compartments (d1, d2, d3, and d4) (Figure 1B). NaT, NaP, K(DR), K(AHP), and H were included in the somatic compartment, with an L-type calcium current (CaL) added to the dendritic compartments (d1 and d2). Although CaL appears to be expressed in both the soma and proximal dendrites of spinal motoneurons in mouse (Carlin et al., 2000a), turtle (Booth et al., 1997), and many other vertebrate motoneurons, its principal contributions to motoneuron behaviors including bi-stable firing (Carlin et al., 2000b) and the amplification of synaptic inputs (Binder et al., 2020) are mainly mediated by its presence in the dendrites. For this reason, we restricted CaL to the dendrite compartments in this study.

**TABLE 1:** Membrane properties of hypoglossal motoneuron.

Properties	Experiment	Model 1	Model 2	Model 3	Model 4	Model 5
RMP (mV)	−70 ± 1	−70.2	−69.0	−70.5	−69.5	−71.0
R_in_ (MΩ)	34 ± 1.7	32.8	34.8	34.0	35.2	33.5
Rheobase (nA)	0.5 ± 0.1	0.49	0.4	0.55	0.51	0.47
V_th_(mV)	−45 ± 8	−51.8	−49.1	−48.5	−47.9	−45.8
AHP duration (ms)	110 ± 3	107	108	110	104	112
AHP amplitude (mV)	−6.1 ± 0.3	−5.7	−6.2	−6.0	−6.1	−6.3
AP width (ms)	3.5 ± 0.5	3.4	4.0	3.8	3.6	3.0

Note: The rheobase and V_th_ were measured from spikes induced by current pulse.

**TABLE 2:** Morphological parameters of hypoglossal motoneuron.

Compartments	Length (μm)	Diameter (μm)	RM (Ωcm^2^)	RA (Ωcm)	CM (μF/cm^2^)
Model 1					
Soma	10	10	1,000	30	1
d1-d2	70	30	8,000	30	1
d3-d4	60	20	8,000	30	1
Model 2					
Soma	30	20	1,100	35	1
d1-d2	70	30	8,000	35	1
d3-d4	70	40	8,000	35	1
Model 3					
Soma	25	10	980	40	1
d1-d2	80	50	8,300	40	1
d3-d4	60	30	8,300	40	1
Model 4					
Soma	15	15	1,000	45	1
d1-d2	80	30	7,000	45	1
d3-d4	80	30	7,000	45	1
Model 5					
Soma	30	30	1,100	50	1
d1-d2	90	50	7,000	50	1
d3-d4	90	50	7,000	50	1

The conductances assigned to the different ionic channels in our models are specified in Table 3. The current equation for the HG motoneuron model was given by

$$C_{m}\frac{dV_{m}}{dt}=-I_{ionic}+I_{inject}$$ (2)

where *C*_*m*_, *I*_*ionic*_ *,* and *I*_*inject*_ were membrane capacitance, ionic currents, and injected currents, respectively. Conductances were set as shown in Table 3. Ionic currents include

INaT=gNaT⋅m3⋅h⋅(Vm−ENa)INaP=gNaP⋅m⋅s⋅(Vm−ENa)IK(DR)=gK(DR)⋅n4⋅(Vm−EK)IK(AHP)=gK(AHP)⋅q⋅(Vm−EK)IH=gH⋅m⋅H(Vm−EH)ICaL=gCaL⋅mCaL⋅(Vm−ECa)ILeak=gLeak(Vm−ELeak) (3)

where *g*_*NaT*_ was the maximum conductance for fast Na^+^ current (*I*_*NaT*_), *g*_*NaP*_ for persistent Na^+^ current (*I*_*NaP*_), *g*_*K(DR)*_ for delayed-rectifier K^+^ current [*I*_*K(DR)*_], *g*_*K(AHP)*_ for CaL-dependent K^+^ current [*I*_*K(AHP)*_], g_CaL_ for CaL current (*I*_*CaL*_), g_h_ for h-current (*I*_*h*_), and *g*_*Leak*_ for leak current (*I*_*Leak*_). *E*_*Na*_, *E*_*K*_, *E*_*Ca*_, *E*_*H*_, and *E*_*Leak*_ were equilibrium potentials for Na^+^, K^+^, Ca^2+^, H, and Leak currents and were set to 55 mV, −75 mV, 80 mV, −55 mV, and −70 mV, respectively. The resting membrane potential (RMP) of the model cells was set to −70 mV. State variables *m, h, n*, and *q* were defined by Hodgkin-Huxley equations, and detail of these parameters were summarized in Supplementary Appendix.

**TABLE 3:** Distribution and density of ionic conductances (mS/cm^2^).

Compartments	NaT	NaP	K(DR)	K (AHP)	CaL	Leak
Model 1						
Soma	320	18	150	10	—	1
d1-d2	—	—	—	—	10	0.13
d3-d4	—	—	—	—	—	0.13
Model 2						
Soma	300	20	140	15	—	0.9
d1-d2	—	—	—	—	12	0.13
d3-d4	—	—	—	—	—	0.13
Model 3						
Soma	270	18	120	11	—	1.02
d1-d2	—	—	—	—	13	0.12
d3-d4	—	—	—	—	—	0.12
Model 4						
Soma	280	17	110	12	—	1
d1-d2	—	—	—	—	12	0.14
d3-d4	—	—	—	—	—	0.14
Model 5						
Soma	260	15	120	11	—	0.9
d1-d2	—	—			14	0.14
d3-d4	—	—	—	—	—	0.14

The voltage-gated opening and closing properties of membrane channels generally become more sensitive to the changes in membrane potential when external stimuli or drugs are applied (Dai et al., 2009; MacDonell et al., 2015). Here, we superimposed a sine function on the maximum conductance of the ion channel models to amplify the channel-mediated currents. This simple addition effectively overrides the smooth and continuous values inherent in the standard Hodgkin-Huxley formulations to simulate the stochasticity of channel flicker (Dorval, 2006). The channel flicker was defined as:

$$g_{CF}=g_{ionic}+a\cdot \sin\left(b\cdot t\right)$$ (4)

where $g_{CF}$ included a sinusoidal function with amplitude *a* and angular frequency *b* and the maximum conductance $g_{ionic}$ . *a* and *b* were adjustable parameters independent in each channel. The amplitude of channel flicker could be changed by adjusting *a* within range of 0.02–0.2 mS and *b* of 0.1–0.2. In general, a membrane channel with CF was expressed as:

$$I_{ionic}=g_{CF}\cdot m^{p}\cdot h^{q}\cdot \left(V_{m}-E_{ionic}\right).$$ (5)

The instantaneous firing frequency (*f*) was measured as reciprocal of time interval between two action potentials. We measured the time (*t*_i_) from the voltage threshold of the ith action potential and calculated the f between two consecutive action potentials by formula: *f* = 1/(*t*_i_-*t*_i-1_). The Clampfit (10.7) provides users with a tool to calculate the instantaneous firing frequency of repetitive firing.

Figures 1C,D show a family of bi-ramp currents with a duration of 6 s and a step of 0.2 nA ramp peak that were injected into a HG motoneuron (Figures 1C1,C2) and HG model (Figures 1D1,D2), respectively. As in this example, repetitive firing induced by the current ramp with a 0.8 nA ramp peak was observed in both HG motoneuron and model without (Figures 1C1,D1) or with white noise (Figures 1C2,D2). Figures 1C3,C4 show the instantaneous firing frequency of the motoneuron without (C3) and with white noise (C4) during the ascending phase of the ramp, which demonstrate that the noise substantially increased discharge variability. Similar results were obtained from the model (Figures 1D3,D4). The white noise superimposed by current transients (top traces in Figures 1C2,D2) increased the firing frequency as well as variability of the firing in both HG motoneuron and model. In this study, a family of bi-ramp currents with increased ramp peaks was injected into the motoneurons. The relationship between slopes of rising phase of the ramps and the step of ramp peaks were established in Figure 1E1, and the number of cells (*n* = 59) recorded at each step of the ramps in both control and white noise were summarized in Figure 1E2. Accordingly, the frequency distribution of the firing at each step was calculated in control (Figure 1E3) and white noise added conditions (Figure 1E4), which showed that the noise dramatically increased the discharge rates, even during the downward phase of the ramps. In this study, we routinely used current ramps with different peaks to explore the dynamic characteristics of repetitive discharge. We collected data on the firing properties of 59 HG motoneurons, including voltage threshold (V_th_), recruitment current (I_rec_), decruitment current (I_dec_), and the difference between decruitment current and recruitment current (ΔI = I_dec_–I_rec_). We constructed five, different HG motoneuron models to accommodate the dynamic range of the firing properties observed in different HG motoneurons. This permitted us to make detailed, statistical comparisons of how effectively our model motoneurons reproduced the full range of HG motoneuron behaviors (Table 1).

## 3 Results

### 3.1 Effect of white noise on voltage threshold

**FIGURE 2:**
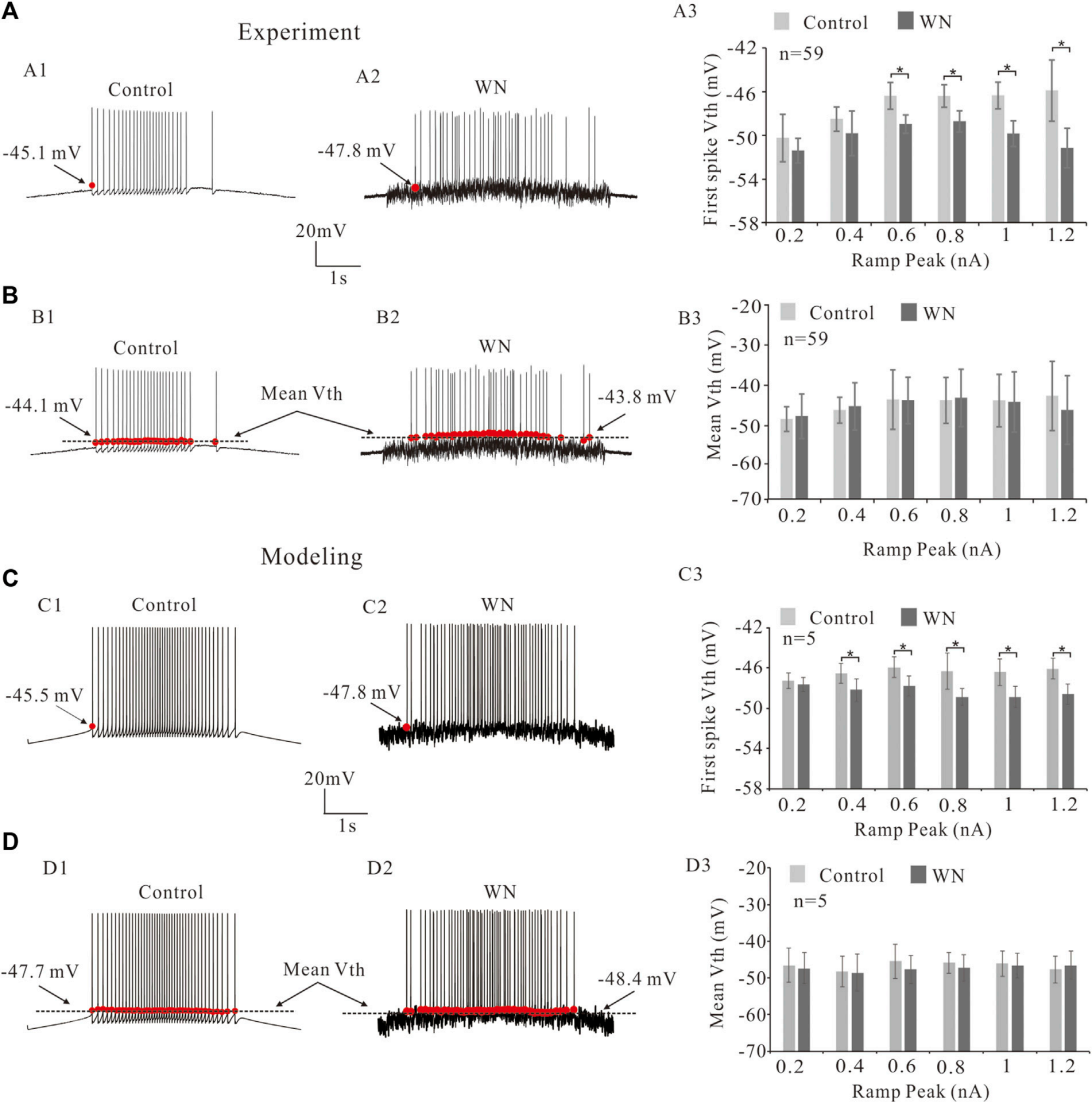
Effect of white noise on voltage threshold (V_th_). **(A)** Repetitive firings were induced by current ramps in control **(A1)** and white noise **(A2)**. White noise hyperpolarized the V_th_ for the first spike (red dots in **A1,A2**). Statistical results showed that the white noise significantly reduced V_th_ for the first spikes from ramp peaks of 0.6–1.2 nA (*p* < 0.05, A3). **(B)** The effects of white noise on the mean values of V_th_ for all spikes (black lines in **B1,B2**). Statistical results showed that white noise did not significantly reduce the mean values of V_th_ **(B3)**. **(C)**: Modeling results showed that white noise significantly reduced the first spike V_th_ (red dots in **C1,C2**) from ramp peaks of 0.4–1.2 nA (*p* < 0.05). **(D)**: Statistical results **(D3)** showed that there was no significant change in the mean values of V_th_ for all spikes (black lines in **D1,D2**). Data are expressed as mean ± SD. Paired *t*-test was performed. *p < 0.05.

The excitability of spinal motoneurons can be regulated by altering the voltage threshold (V_th_) for spike initiation. In general, this V_th_ alteration is related to the modulation of voltage-gated channels such as NaT and/or K(DR) (Krawitz et al., 2001; Dai et al., 2002; MacDonell et al., 2015; Dai et al., 2018). Figure 2A shows that injected white noise (WN) hyperpolarized the V_th_ for the first spike by about 3 mV (Figures 2A1,A2, red dots). Statistical analysis of the results derived from 59 HG motoneurons showed that significant hyperpolarization of V_th_ for the first spike was observed within the range of the ramp peaks from 0.6 to 1.2 nA. This WN-induced V_th_ hyperpolarization appeared to be slope-dependent, in that the higher the ramp peak, the larger the V_th_ hyperpolarization (Figure 2A3). However, there was no significant difference in the mean values of V_th_ for all spikes between control (Figure 2B1, dash line) and WN data (Figures 2B2,B3). These results were well replicated by our HG motoneuron models (Figure 2C,D). The simulation results showed that WN hyperpolarized the V_th_ for the first spike (Figures 2C2,C3) and that there was no significant change in the mean values of V_th_ for all spikes recorded with WN (Figures 2D2,D3).

### 3.2 Effect of white noise on recruitment and decruitment currents

**FIGURE 3:**
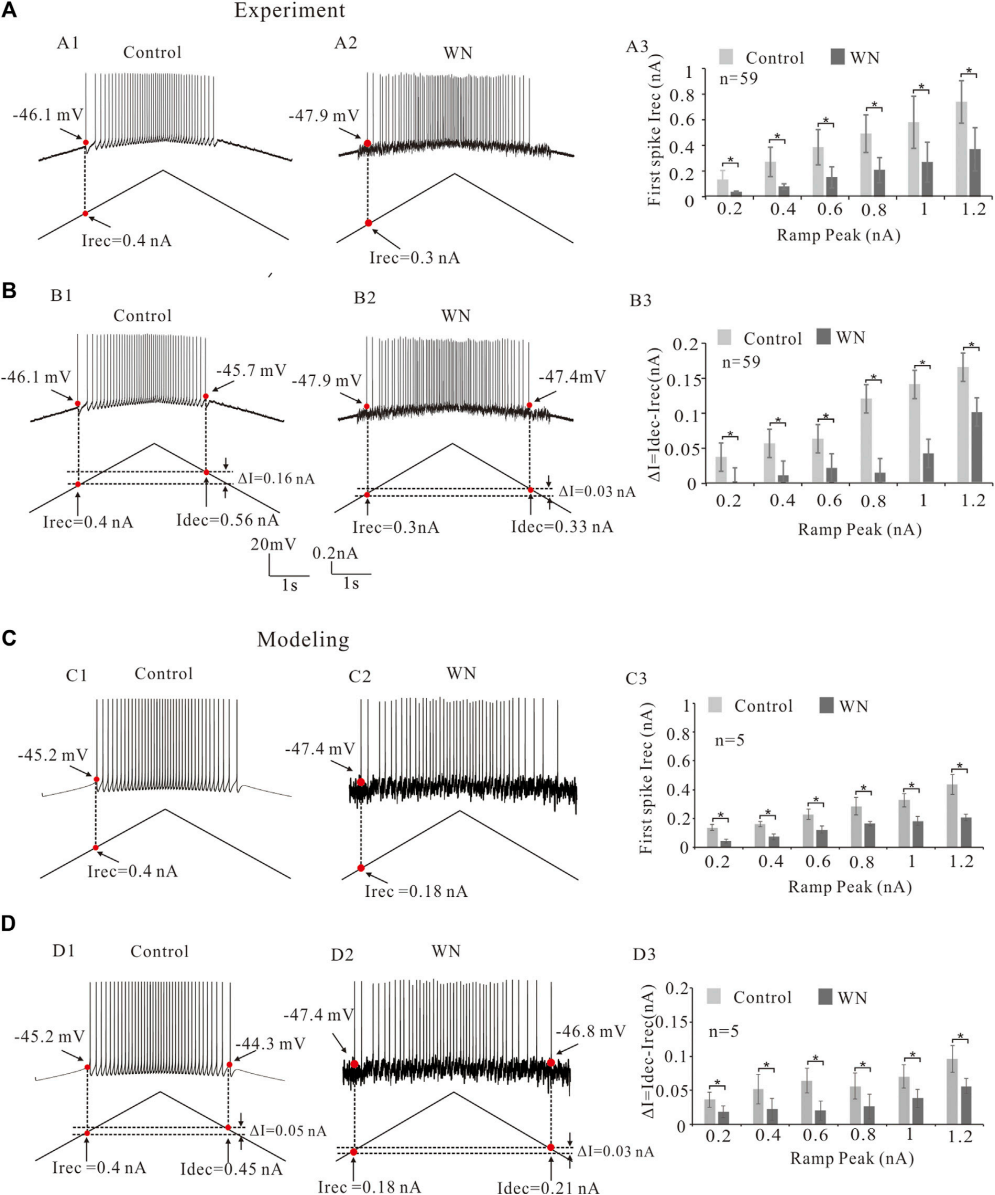
Effect of white noise on recruitment current (I_rec_). **(A)** Repetitive firings were evoked by current bi-ramps in control **(A1)** and white noise **(A2)** and current thresholds (I_rec_) were recorded (red dots). White noise reduced the I_rec_ (**A1** vs. **A2**). Statistical results showed that the white noise significantly reduced the I_rec_ of the repetitive firing and this reduction of I_rec_ was slope-dependent **(A3)**. **(B)** Recruitment, decruitment currents (red dots in **B1,B2**) and ΔI = I_dec_- I_rec_ were calculated. White noise reduced ΔI (**B1** vs. **B2**). Statistical results indicated that the white noise significantly reduced ΔI **(B3)**. The reduction of ΔI was slope-dependent. **(C)** Similar results were obtained from modeling data. Similarly, repetitive firings were generated by current bi-ramps in control **(C1)** and white noise **(C2)**. Recruitment current (I_rec_) were recorded (red dots). White noise reduced the I_rec_ (**C1** vs. **C2**). Statistical results indicated that white noise significantly reduced the I_rec_ **(C3)**. The reduction of I_rec_ was also slope-dependent. **(D)**: Recruitment, decruitment currents (red dots in **D1** and **D2**) and ΔI = I_dec_- I_rec_ were calculated from the repetitive firings in control **(D1)** and white noise **(D2)**. White noise reduced ΔI (**D1** vs. **D2**). Statistical results showed that the white noise significantly reduced ΔI **(D3)**, and the reduction of ΔI was shown to be slope-dependent. Data are expressed as mean ± SD. Paired *t*-test was performed. *p < 0.05.

Neuronal excitability is generally characterized not only by voltage threshold, but also by recruitment (I_rec_) and decruitment (I_dec_) currents (e.g., Cheng et al., 2020; Cheng et al., 2021). In the present study, we defined the recruitment current as the current ramp at which the first spike was initiated (Figures 3A1,A2, red dots) and decruitment current as the current ramp at which the last spike was elicited (Figures 3B1,B2). We further defined ΔI = I_dec_–I_rec_ which provides an estimate of the magnitude of persistent inward currents (PICs) (Gorassini et al., 2002). Our analysis showed that white noise significantly reduced the I_rec_ (Figures 3A1,A2) and that this reduction of I_rec_ appeared to be ramp peak-dependent (slope-dependent), i.e., the steeper the slope of the ramp, the larger the amount of I_rec_ reduction (Figure 3A3; *n* = 59, *p* < 0.05). The white noise also lowered ΔI, prolonging the duration of repetitive firing (Figures 3B1,B2). Similarly, a ramp peak-dependent lowering of ΔI was observed (Figure 3B3; *n* = 59, *p* < 0.05). These results were mimicked by our models for both I_rec_ (Figures 3C1,C2) and ΔI with white noise (Figures 3D1,D2). Statistical results confirmed that white noise reduced I_rec_ and ΔI (Figures 3C3,D3; *n* = 5, *p* < 0.05), and furthermore the reduction demonstrated a dependence on the slope of the current ramp (Figures 3A3,B3).

### 3.3 Effect of white noise on frequency-current relationships

**FIGURE 4:**
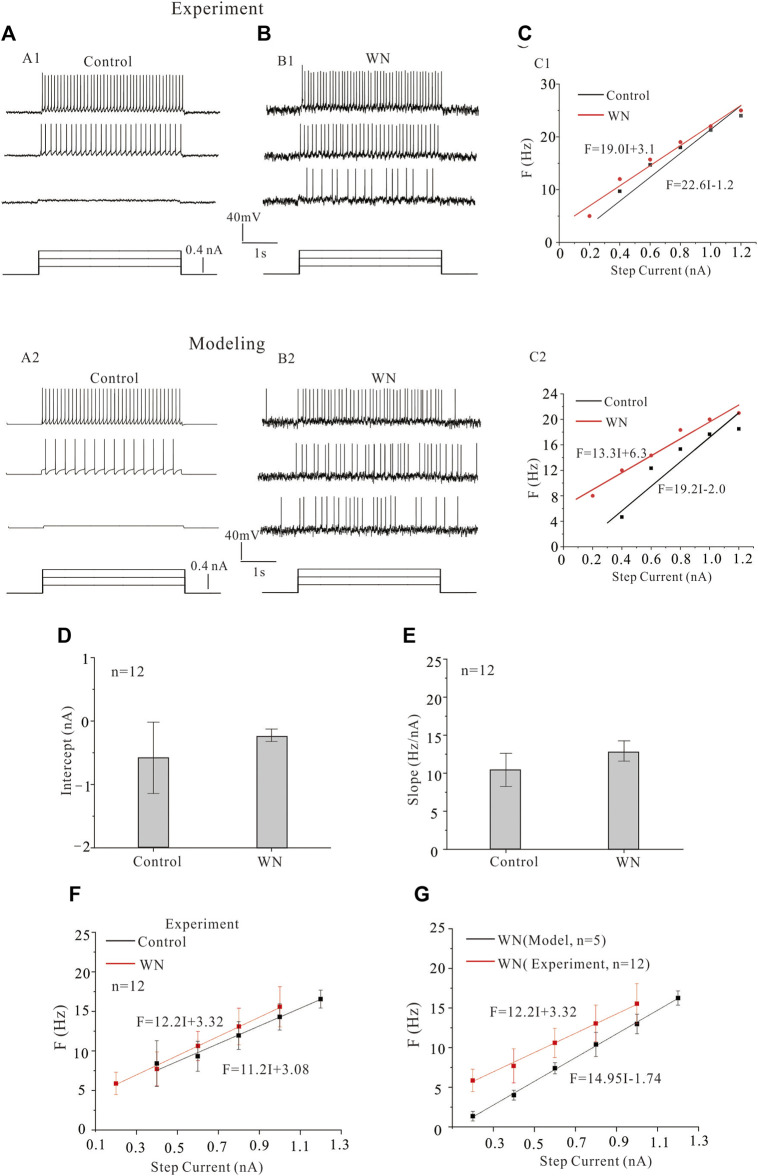
Effect of white noise on frequency-current (F-I) relationships. **(A,B)** Repetitive firing in HG motoneurons was induced by injecting step currents of 3 s duration and 0.2 nA step in both experiments **(A1,B1)** and models **(A2,B2)** recordings without (control) and with white noise, respectively. **(C)** The F-I relationships were measured for the experiment data **(C1)** and modeling results **(C2)**. White noise increased discharge rates and shifted the F-I curve to the left. **(D,E)** Statistical results from 12 motoneurons showed that white noise slightly increased the intercept and slope of the F-I relationship. **(F)** The F-I relationships for control (black line) and white noise (red line) were established from the statistical results **(D,E)**. **(G)** The F-I relationship with WN was established from simulation data of 5 HG motoneuron models (black line), and the F-I curve from the experimental data with WN was plotted in the same diagram to show the difference between the experimental and modeling data recorded with white noise.

In addition to the effects of noise on V_th_ and I_rec_, we also investigated its effect on the F-I relationship. Figure 4 shows the repetitive discharge of an HG motoneurons injected with a 0.2 nA current step with a 3 s duration in both control (Figure 4A) and white noise (Figure 4B) conditions. The F-I relationships were measured in both conditions (Figure 4C) to demonstrate the noise-modulated output of HG motoneurons. The experimental data are shown in Figures 4A1–C1) and the modeling results in Figures 4A2–C2). It is clear that the white noise increased the discharge rates and shifted the F-I curves to the left with a 3.6 Hz/nA and 5.9 Hz/nA reduction of the slopes in the experiment data (Figure 4C1) and modeling results (Figure 4C2), respectively. Statistical analysis of the data from 12 HG motoneurons showed that the white noise did not significantly alter the F-I curves in both intercept (Figure 4D, intercept: *p* = 0.23) and slope (Figure 4E, slope: *p* = 0.08). The F-I curves showed that white noise reduced the minimum current for repetitive firing with little change in the slope (Figure 4F).

We attempted to reproduce the experimental F-I curves we observed with our HG motoneuron models. Statistical results from 5 HG motoneuron models (Tables 1–3) demonstrated that the WN-induced F-I curve of the models was significantly different from that of experimental F-I curve in both slope (14.95 vs. 12.2 Hz/nA, *p* = 0.001) and intercept (Figure 4G, 0.12 vs. −0.27 nA, *p* = 0.007). The failure of our models to completely reproduce our experimental results suggested that the models required additional intrinsic mechanisms to account for the effects of subthreshold membrane potential oscillations on excitability. As a consequence, we introduced modifications to the kinetics of voltage-gated channels in the model that were functionally equivalent to the noise generated by the stochastic flicker of voltage-gated ion channels (channel flicker, CF, White et al., 2000; Dorval and White, 2005; Dorval, 2006) as described below.

### 3.4 Effect of channel flicker on the F-I relationships

**FIGURE 5:**
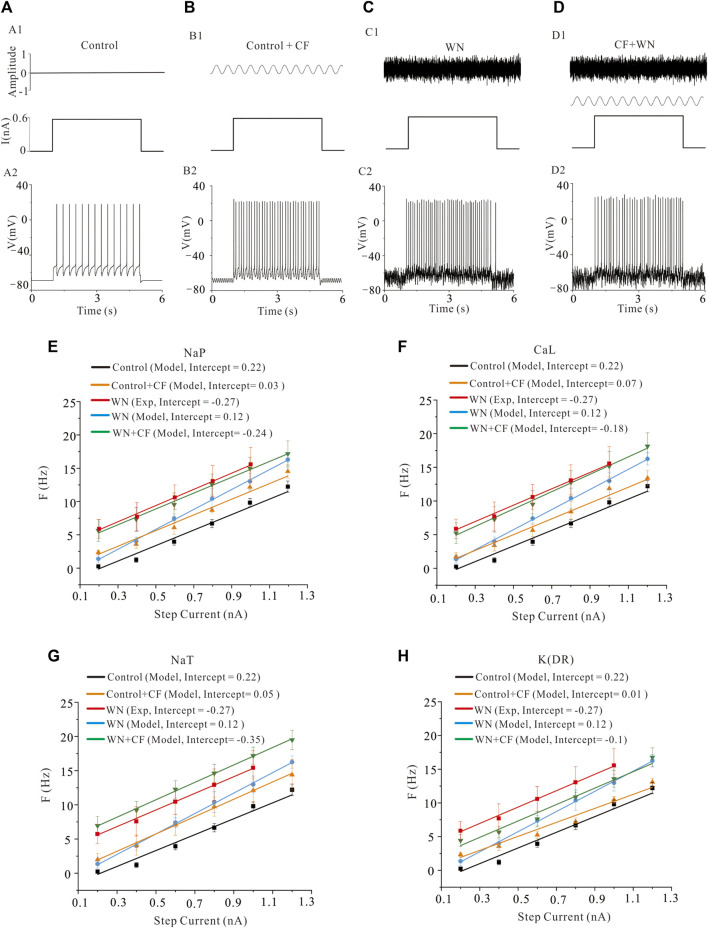
Effect of channel flicker (CF) on the F-I relationships. **(A)** Repetitive firing **(A2)** was induced by step currents **(A1)**. **(B)** Repetitive firing **(B2)** induced by the same step currents with CF (**B1**, CF on top, step current on bottom). **(C)**: Repetitive firing **(C2)** induced by the same step currents with white noise (**C1**, WN on top, step current on bottom). **(D)**: Repetitive firing **(D2)** induced by the step currents with combination of white noise and CF **(D1)**. The WN was shown on top, step current and channel flicker waveform on bottom. Note: CF was added to kinetics of ionic channels rather than injected currents. **(E)**: F-I relationships were established in five conditions: rat experimental control data (red curve), model control (black curve), model with NaP channel flicker (orange curve), model with white noise (blue curve) and model with white noise plus CF added to NaP channel (green curve). Modeling results showed that the F-I curve with WN + CF (NaP) (green) closely approximated that obtained from the rat experimental data with WN (red). **(F)**: Similar to the F-I curves in E, five types of F-I curves were established as those in E with only difference in CaL. CF was added to CaL channel (CaL + CF) and the corresponding F-I curve was established in white noise condition. Simulation results indicated that the F-I curve with WN + CF (CaL) (green) closely approximated that obtained from the rat experimental data recorded with WN (red). **(G)**: The same as F-I curves in F, five different F-I curves were established as those in F with only difference in NaT. CF was added to NaT channel (NaT + CF) and the corresponding F-I curve was established with WN. Simulation results showed that the F-I curve with WN + CF (NaT) (green) approached to the experimental data with WN (red). However, the extent of approximation to experimental F-I curve was not as good as NaP and CaL. **(H)** CF was added to K(DR) channel (K(DR)+CF) and F-I curves were calculated under WN condition. Simulation results showed that the F-I curve recorded with WN and WN + CF (K(DR)) was closer to the experimental F-I curve, but a substantial difference between them remained.

A channel flicker (CF) component was added to the HH equations of NaP, K(DR), NaT, and CaL, respectively. Figures 5A−D demonstrates the repetitive firings induced by step currents in four conditions: control (Figure 5A1), control plus CF (Figure 5B1, CF: sine waveform), white noise (Figure 5C1), and white noise plus CF (Figure 5D1) in the models. The F-I curves with WN were established with CF introduced to NaP (Figure 5E, green curves), CaL (Figure 5F, green curves), NaT (Figure 5G, green curves), and K(DR) (Figure 5H, green curves), respectively. To facilitate comparisons, we added several other F-I curves to the figures, which were obtained from the rat experiments (red curves), a control model (black curves), a model with CF (orange curves), and a model with white noise (blue curves). The simulation results indicated that with channel flicker, the F-I curves more closely approximated those obtained from the rat experiments with WN. Among the four channels, modulating the CaL and NaP produced the closest approximation to the experimental F-I curves, followed by NaT and K(DR) in terms of F-I slopes and intercepts. The difference between experimental and modeling F-I curves included the slope of −0.31 Hz/nA and intercept of 0.03 nA for NaP, 0.7 Hz/nA and 0.09 nA for CaL, 0.5 Hz/nA and −0.08 nA for NaT, and −0.02 Hz/nA and 0.17 nA for K(DR), respectively. We found that adding the channel flicker terms substantially improved the models’ capacity to reproduce the experimental F-I curves obtained with white noise.

### 3.5 Effect of channel flicker on voltage threshold

**FIGURE 6:**
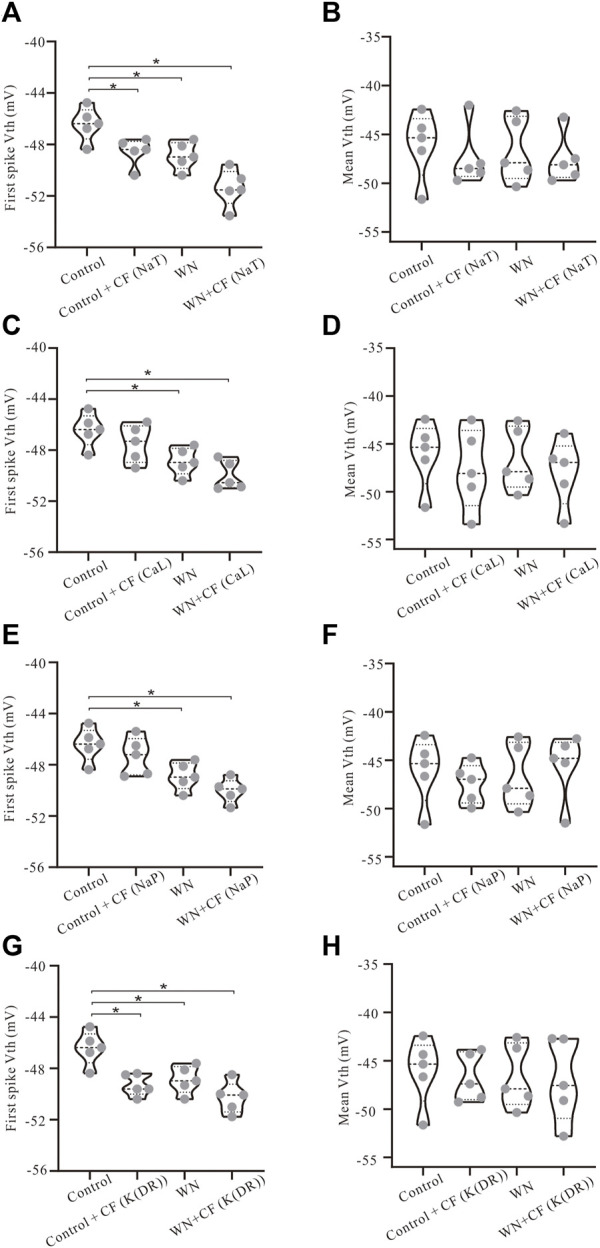
Effect of channel flicker on voltage threshold. The simulation data presented below were recorded with current ramp peak of 1 nA. **(A)** V_th_ for the first spike was hyperpolarized in Control + CF (NaT), WN and WN + CF (NaT). **(B)** No significant hyperpolarization of V_th_ was observed in mean values of V_th_ for all spikes. **(C)** V_th_ for the first spike was hyperpolarized in both WN and WN + CF (CaL). **(D)** No significant hyperpolarization was obtained in mean values of V_th_ for all spikes in Control + CF (CaL), WN and WN + CF (CaL). **(E)** V_th_ for the first spike was lowered in both WN and WN + CF (NaP). **(F)** There was no substantial hyperpolarization of mean values of V_th_ in Control + CF (NaP), WN and WN + CF (NaP). **(G)** Control + CF (K(DR)), WN and WN + CF (K(DR)) induced the significant lowering of V_th_ for the first spike. **(H)** K(DR)+CF did not significantly alter the mean V_th_ for all spikes with or without WN. *p < 0.05.

The introduction of channel flicker (CF) to the kinetics of the channels in our models generated F-I relationships that much more closely mimicked the experimental results. However, the effects of channel flicker on V_th_ and I_rec_ remained unclear. We explored this issue with additional simulations. To simplify our presentation, we have selected data recorded with ramp peak of 1 nA in Figure 6 (Additional results collected with other ramp peaks from 0.2 to 1.2 nA are summarized in Table 4). Firstly, we added CF to NaT, and found that Control + CF, WN, and WN + CF induced the hyperpolarization of V_th_ for the first spike of repetitive firing (Figure 6A, *p* < 0.05). Moreover, the amount of V_th_ hyperpolarization induced by WN + CF was bigger than those by CF or WN individually. However, no significant hyperpolarization of V_th_ was obtained in the mean values of V_th_ for all spikes (Figure 6B), similar to what we found in the experimental results (Figures 2A,B). Next, we then added CF to CaL. The simulation results indicated that WN + CF hyperpolarized V_th_ for the first spike, but no significant hyperpolarization of V_th_ was shown in Control + CF (Figure 6C). Again, the hyperpolarization was not obtained in the model for the mean values of V_th_ for all spikes generated (Figure 6D). We further examined the effect of NaP with CF on the V_th_. Similar to our results in modifying CaL, white noise or white noise with NaP + CF induced a significant hyperpolarization of V_th_ for the first spike (Figure 6E, *p* < 0.05). However, no substantial hyperpolarization of V_th_ was obtained in the mean values of V_th_ for all spikes (Figure 6F). Finally, we explored the effects of K(DR) with CF on V_th_. The hyperpolarization of V_th_ for the first spike was induced by Control + CF, WN, and WN + CF (Figure 6G, *p* < 0.05), and this hyperpolarization was not observed in mean values of V_th_ for all spikes recorded (Figure 6H). The above results indicated that the response of HG motoneurons to WN-induced membrane oscillation do not show significant differences between the channels with and without CF in terms of V_th_ hyperpolarization for the first spike. However, the amount of the V_th_ hyperpolarization induced by WN + CF was always bigger than that by WN alone within the entire range of ramp peaks, demonstrating the contribution of CF to the V_th_ hyperpolarization. Moreover, NaT and K(DR) with CF could consistently hyperpolarize V_th_ for the first spike without white noise, suggesting that the CF could make the motoneurons more sensitive to subthreshold membrane potential for spike generation.

**TABLE 4:** Effect of channel flicker on voltage threshold.

Ramp peak (nA)		First spike V_th_ (mV)				Mean V_th_ (mV)			
		NaT	CaL	NaP	K(DR)	NaT	CaL	NaP	K(DR)
0.2	Control		−47.3 ± 0.8				−46.5 ± 4.6		
	Control + CF	−48.9 ± 1.0	−47.6 ± 0.9	−48.2 ± 1.0	−48.9 ± 0.5	−47.8 ± 3.3	−46.2 ± 3.8	−47.2 ± 3.6	−46.9 ± 3.0
	WN		−47.6 ± 0.7				−47.4 ± 4.3		
	WN + CF	−49.6 ± 1.8	−49.1 ± 1.4	−48.3 ± 0.7	−49.2 ± 0.8	−47.6 ± 2.5	−45.9 ± 3.3	−45.9 ± 4.3	−47.0 ± 3.6
0.4	Control		−46.6 ± 1.0				−48.3 ± 4.2		
	Control + CF	−48.5 ± 0.7	−47.2 ± 1.4	−47.7 ± 1.7	−49.1 ± 0.5	−48.7 ± 2.3	−47.6 ± 3.2	−46.7 ± 2.8	−47.1 ± 2.9
	WN		−48.2 ± 1.1				−48.6 ± 5.1		
	WN + CF	−50.1 ± 1.6	−49.0 ± 1.1	−49.4 ± 1.4	−48.6 ± 1.1	−48.5 ± 2.6	−48.1 ± 2.9	−45.0 ± 3.7	−46.5 ± 3.3
0.6	Control		−45.9 ± 1.0				−45.5 ± 4.6		
	Control + CF	−48.9 ± 1.0	−47.6 ± 0.9	−47.0 ± 1.1	−48.2 ± 1.1	−47.0 ± 2.8	−46.4 ± 4.1	−46.0 ± 3.5	−46.8 ± 3.3
	WN		−47.8 ± 1.0				−47.7 ± 3.8		
	WN + CF	−49.4 ± 1.8	−49.2 ± 1.5	−48.3 ± 0.9	−49.2 ± 0.7	−45.5 ± 3.5	−46.7 ± 3.6	−45.5 ± 4.0	−46.3 ± 3.0
0.8	Control		−46.3 ± 1.8				−45.9 ± 2.9		
	Control + CF	−49.1 ± 1.0	−47.5 ± 0.9	−48.1 ± 1.5	−49.0 ± 0.9	−47.1 ± 2.9	−46.2 ± 3.4	−45.9 ± 2.7	−47.2 ± 2.8
	WN		−48.9 ± 0.8				−47.3 ± 3.6		
	WN + CF	−50.5 ± 1.0	−49.5 ± 1.1	−49.6 ± 1.4	−49.9 ± 1.5	−46.5 ± 3.3	−46.6 ± 4.3	−45.8 ± 3.3	−46.1 ± 3.6
1.0	Control		−46.4 ± 1.3				−46.1 ± 3.5		
	Control + CF	−48.6 ± 1.1	−47.5 ± 1.5	−47.3 ± 1.5	−49.4 ± 0.9	−47.4 ± 3.1	−47.6 ± 4.2	−47.3 ± 3.0	−46.8 ± 3.3
	WN		−48.9 ± 1.1				−46.6 ± 3.3		
	WN + CF	−51.4 ± 1.5	−50.0 ± 1.1	−50.0 ± 0.9	−50.3 ± 1.2	−47.5 ± 2.6	−48.0 ± 3.5	−45.6 ± 3.5	−47.0 ± 4.3
1.2	Control		−46.1 ± 1.0				−48.3 ± 3.1		
	Control + CF	−48.3 ± 0.8	−47.3 ± 1.3	−47.7 ± 1.4	−49.3 ± 0.8	−48.3 ± 3.1	−46.4 ± 3.1	−46.1 ± 3.3	−46.9 ± 2.9
	WN		−48.6 ± 1.0				−46.6 ± 3.9		
	WN + CF	−52.2 ± 1.7	−50.5 ± 0.7	−50.6 ± 1.3	−50.0 ± 1.3	−46.3 ± 2.3	−46.5 ± 4.2	−46.7 ± 3.9	−45.6 ± 4.2

### 3.6 Effect of channel flicker on recruitment and decruitment currents

**FIGURE 7:**
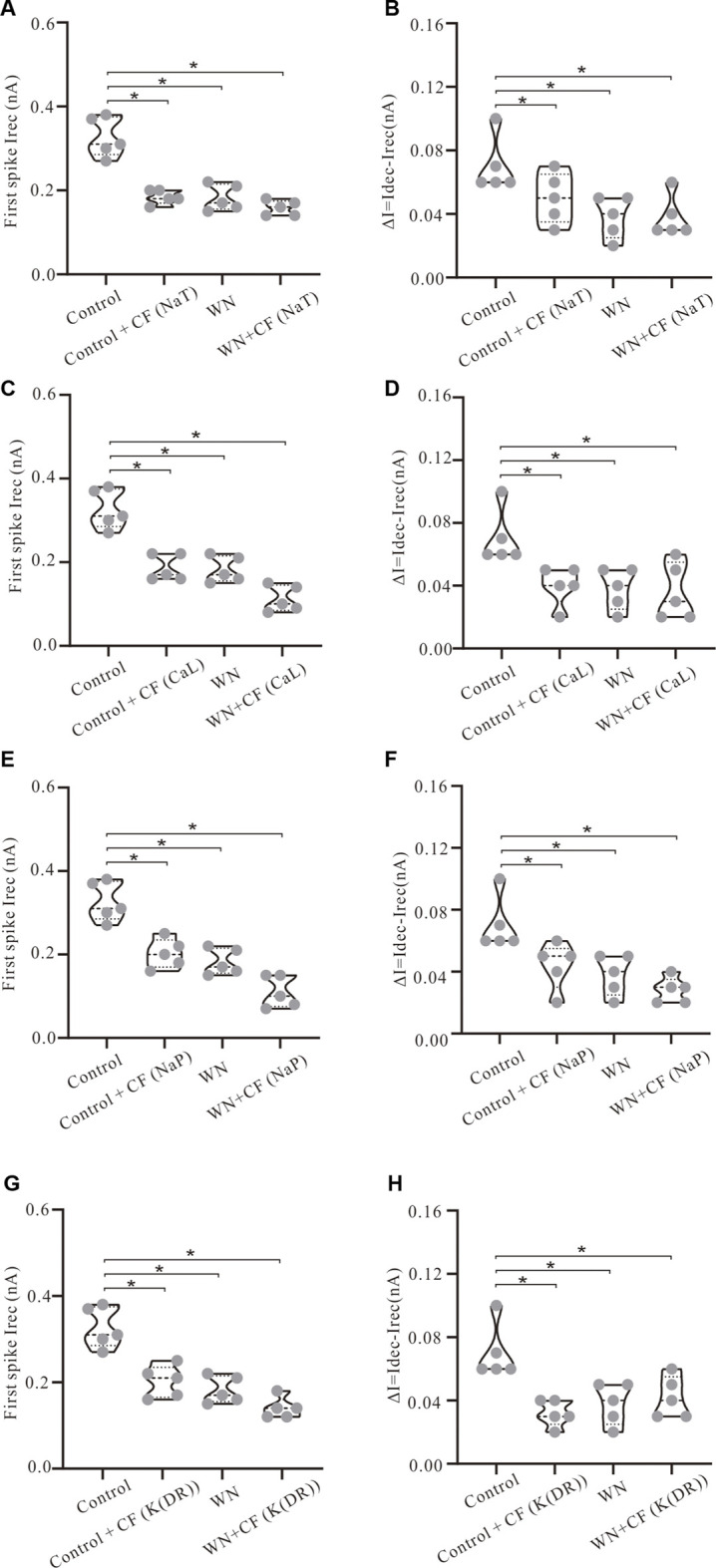
Effect of channel flicker (CF) on recruitment and decruitment currents. The simulation data presented below were recorded with current ramp peak of 1 nA. **(A)** I_rec_ was significantly reduced in Control + CF (NaT), WN and WN + CF (NaT). **(B)** ΔI was reduced significantly in Control + CF (NaT), WN and WN + CF (NaT). **(C)** I_rec_ was significantly reduced in Control + CF (CaL), WN and WN + CF (CaL) and the amount of reduction of I_rec_ induced by WN + CF (CaL) was bigger than that by Control + CF (CaL) or WN. **(D)** ΔI was reduced significantly in Control + CF (CaL), WN and WN + CF (CaL). **(E)** I_rec_ was significantly reduced in Control + CF (NaP), WN and WN + CF (NaP), and the amount of I_rec_ reduction induced by WN + CF (NaP) was bigger than that by Control + CF (NaP) or WN. **(F)** ΔI was reduced significantly in Control + CF (NaP), WN and WN + CF (NaP). **(G)** I_rec_ was significantly reduced in Control + CF (K(DR)), WN and WN + CF (K(DR)). The amount of reduction of I_rec_ induced by WN + CF (K(DR)) was bigger than that by Control + CF (K(DR)) or WN. **(H)** ΔI was reduced significantly in Control + CF (K(DR)), WN and WN + CF (K(DR)). *p < 0.05.

Channel flicker (CF) not only affected voltage threshold, but also recruitment and decruitment currents. The simulation results showed that white noise induced significant changes in I_rec_ and ΔI when CF was added to the channels. In Figure 7, we selected data recorded with ramp peak of 1 nA (Again, additional results collected with other ramp peaks from 0.2 to 1.2 nA are summarized in Table 5.) We first studied the effect of NaT + CF on I_rec_ and ΔI. Our simulations showed that I_rec_ was reduced in Control + CF, WN, and WN + CF conditions (Figure 7A, *p* < 0.05). Further analysis showed that the amounts of reduction of I_rec_ by WN + CF were bigger than those by CF or WN individually, suggesting that less I_rec_ was required to make the cell discharge when NaT + CF was activated by white noise. We further explored the effect of CF on ΔI with similar outcomes (Figure 7B). Compared with the control, Control + CF, WN, and WN + CF all reduced ΔI, suggesting that NaT + CF prolonged the repetitive firing of HG motoneurons.

**TABLE 5:** Effect of channel flicker on recruitment and decruitment current.

Ramp peak (nA)		I_rec_ (nA)				ΔI (nA)			
		NaT	CaL	NaP	K(DR)	NaT	CaL	NaP	K(DR)
0.2	Control		0.14 ± 0.018				0.04 ± 0.011		
	Control + CF	0.04 ± 0.011	0.03 ± 0.01	0.04 ± 0.01	0.03 ± 0.012	0.02 ± 0.012	0.03 ± 0.011	0.02 ± 0.011	0.02 ± 0.007
	WN		0.04 ± 0.012				0.02 ± 0.004		
	WN + CF	0.02 ± 0.005	0.02 ± 0.004	0.02 ± 0.008	0.03 ± 0.011	0.02 ± 0.008	0.01 ± 0.002	0.01 ± 0.005	0.02 ± 0.011
0.4	Control		0.16 ± 0.019				0.05 ± 0.022		
	Control + CF	0.09 ± 0.018	0.07 ± 0.024	0.07 ± 0.021	0.07 ± 0.016	0.02 ± 0.005	0.03 ± 0.011	0.03 ± 0.012	0.03 ± 0.016
	WN		0.07 ± 0.02				0.02 ± 0.016		
	WN + CF	0.06 ± 0.019	0.03 ± 0.013	0.05 ± 0.015	0.03 ± 0.013	0.02 ± 0.015	0.01 ± 0.008	0.02 ± 0.008	0.02 ± 0.010
0.6	Control		0.23 ± 0.036				0.06 ± 0.018		
	Control + CF	0.14 ± 0.023	0.12 ± 0.018	0.12 ± 0.029	0.13 ± 0.011	0.02 ± 0.016	0.02 ± 0.013	0.02 ± 0.011	0.02 ± 0.013
	WN		0.12 ± 0.027				0.02 ± 0.014		
	WN + CF	0.08 ± 0.019	0.04 ± 0.015	0.06 ± 0.021	0.09 ± 0.019	0.02 ± 0.017	0.02 ± 0.007	0.02 ± 0.007	0.03 ± 0.023
0.8	Control		0.28 ± 0.063				0.06 ± 0.019		
	Control + CF	0.17 ± 0.025	0.16 ± 0.021	0.17 ± 0.026	0.16 ± 0.03	0.04 ± 0.011	0.03 ± 0.009	0.04 ± 0.013	0.03 ± 0.009
	WN		0.16 ± 0.016				0.03 ± 0.018		
	WN + CF	0.13 ± 0.027	0.07 ± 0.013	0.07 ± 0.023	0.12 ± 0.019	0.02 ± 0.015	0.03 ± 0.013	0.03 ± 0.013	0.04 ± 0.013
1.0	Control		0.33 ± 0.047				0.07 ± 0.019		
	Control + CF	0.18 ± 0.017	0.19 ± 0.031	0.20 ± 0.035	0.20 ± 0.037	0.05 ± 0.016	0.04 ± 0.012	0.04 ± 0.015	0.03 ± 0.008
	WN		0.18 ± 0.031				0.04 ± 0.013		
	WN + CF	0.16 ± 0.018	0.11 ± 0.031	0.11 ± 0.038	0.14 ± 0.024	0.04 ± 0.013	0.04 ± 0.018	0.03 ± 0.008	0.04 ± 0.013
1.2	Control		0.43 ± 0.069				0.10 ± 0.019		
	Control + CF	0.23 ± 0.034	0.22 ± 0.32	0.27 ± 0.040	0.23 ± 0.024	0.06 ± 0.013	0.05 ± 0.026	0.07 ± 0.020	0.05 ± 0.009
	WN		0.21 ± 0.024				0.06 ± 0.011		
	WN + CF	0.18 ± 0.015	0.14 ± 0.036	0.15 ± 0.027	0.16 ± 0.027	0.06 ± 0.016	0.05 ± 0.018	0.04 ± 0.019	0.05 ± 0.016

Next, we examined the effect of CaL + CF on I_rec_ and ΔI. Simulation results showed that I_rec_ was reduced in Control + CF, WN, and WN + CF, and the amounts of reduction of I_rec_ produced by WN + CF were shown to be larger than those by Control + CF (Figure 7C, *p* < 0.05). Similar results were obtained from ΔI (Figure 7D). ΔI was reduced in Control + CF, WN, and WN + CF, and the amounts of reduction of ΔI were similar in the three conditions (Figure 7D, *p* < 0.05).

Based on the simulation results delineated above, we further investigated the effect of NaP + CF on the firing properties of the HG motoneurons. Similar to the results of NaT + CF, adding channel flicker to NaP led to a reduction of I_rec_ (Figure 7E, *p* < 0.05) as well as ΔI (Figure 7F, *p* < 0.5). Moreover, the amounts of reduction of I_rec_ and ΔI induced by WN + CF were bigger than those by CF or WN individually (Figures 7E,F, *p* < 0.05). Interestingly, the reduction of I_rec_ and ΔI produced by WN + CF (NaP) (Figures 7E,F) were generally larger than those by WN + CF (NaT) (Figures 7A,B), suggesting that NaP + CF might play a more essential role in regulating neuronal excitability than NaT + CF in response to noise-generated membrane potential oscillations.

Finally, we studied the effect of adding channel flicker to K(DR) on repetitive firing. As opposed to NaT, NaP, and CaL, K(DR) is an outward current and its role in spike initiation in the presence of membrane potential oscillations was unknown. Introducing channel flicker to K(DR) produced reductions of both I_rec_ (Figure 7G, *p* < 0.05) and ΔI (Figure 7H, *p* < 0.05) in the models. The amounts of reduction of I_rec_ induced by WN + CF were bigger than those by CF or WN individually (Figure 7G, *p* < 0.05). However, the same was not always true for ΔI. The amounts of reduction of ΔI induced by WN + CF were generally smaller than those induced by CF or WN (Figure 7H), suggesting that duration of repetitive firing regulated by K(DR) could be longer than that by K(DR) +CF alone during white noise-induced membrane potential oscillations. Similar to the lowering of V_th_, adding CF to NaT, NaP, CaL, and K(DR) consistently reduced I_rec_ and ΔI for repetitive discharge with or without WN, suggesting that the CF alone could make the motoneurons more sensitive to subthreshold membrane potential fluctuations in generating tonic firing.

### 3.7 Ionic currents with white noise and channel flicker

**FIGURE 8:**
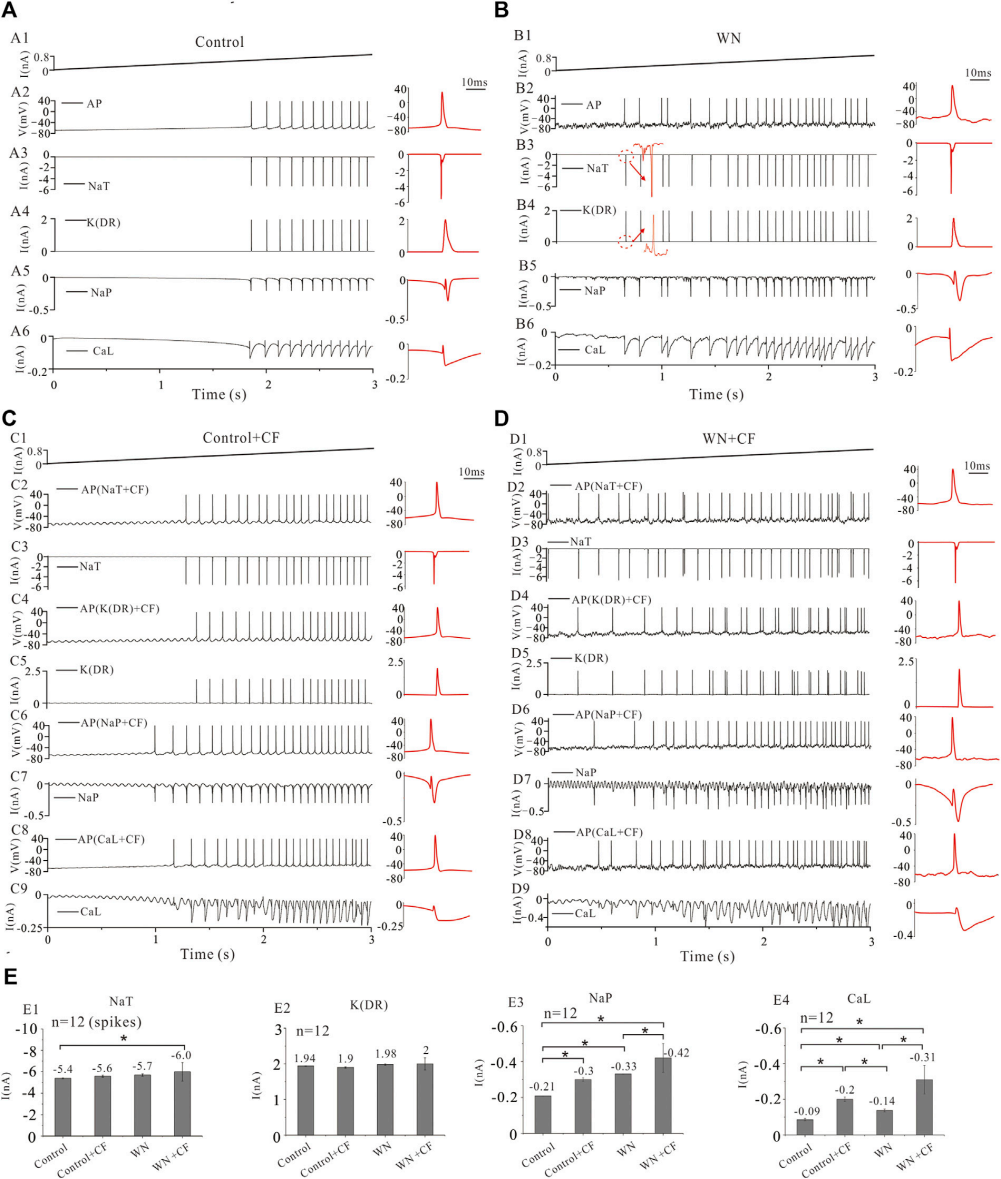
Ionic currents with white noise and channel flicker (CF). **(A)**: Repetitive firings and corresponding currents of NaT, K(DR), NaP, and CaL induced by injection of a ramp current with a duration of 3 s and a peak of 0.8 nA. The red traces in the right insets were action potentials (AP) averaged from the first 12 spikes with averaged corresponding currents. **(B)** Tonic firings were evoked by the same ramp current with white noise. The averaged AP (red) and averaged corresponding currents of NaT, K(DR), NaP, and CaL were shown in the right insets (red traces). More spikes were evoked and larger inward currents were induced by WN. **(C)** The channel flicker (CF) was added to NaT, K(DR), NaP, and CaL, respectively. Repetitive firing was induced by a current ramp **(C1)** and the first 12 spikes were averaged from the firings recorded with NaT + CF **(C2,C3)**, K(DR)+CF **(C4&C5)**, NaP + CF **(C6&C7)** and CaL + CF **(C8&C9)**, respectively. The averaged spikes and averaged ionic currents were shown in the right insets (red traces). More spikes were generated and bigger inward currents were induced by CF. **(D)** The channel flicker (CF) was added to NaT, K(DR), NaP, and CaL, respectively. Repetitive firing was induced by current ramp combined with white noise **(D1)** and the first 12 spikes were averaged from the firings recorded with NaT + CF (D2&D3), K(DR)+CF **(D4&D5)**, NaP + CF **(D6&D7)** and CaL + CF **(D8&D9)**, respectively. The averaged spikes and averaged ionic currents were shown in the right insets (red traces). More spikes were generated and bigger inward currents were induced by WN + CF. **(E)** Statistical results showed that Control + CF (NaT) increased NaT currents by 0.2 nA compared with control (NaT) without CF. WN increased NaT currents by 0.3 nA compared with control. WN + CF (NaT) increased NaT currents by 0.6 nA compared with control **(E1)**. Control + CF (K(DR)), WN, and WN + CF (K(DR)) did not change K(DR) currents significantly **(E2)**. Control + CF (NaP), WN, and WN + CF (NaP) substantially increased the NaP currents **(E3)**. Similar to NaP + CF, Control + CF (CaL), WN, and WN + CF (CaL) also significantly increased CaL currents **(E4)**. Data are expressed as mean ± SD. Unpaired T-tests was performed. *p < 0.05.

Our experimental and simulation results clearly demonstrate that noise-induced membrane potential oscillations alter the output and excitability of HG motoneurons. We further showed that this alteration was related to the modulation of membrane channel behavior, presumably the noise generated by the stochastic flicker of voltage-gated ion channels (CF). Figure 8A shows that tonic firing was evoked by a current ramp with a duration of 3 s and peak of 0.8 nA, and the first 12 action potentials (AP) were averaged (Figure 8A1) with averaged corresponding currents of NaT, K(DR), NaP, and CaL which were responsible for generating the spikes (Figures 8A2–A6, red traces in right panels). With white noise injected into the model, more spikes were elicited (Figure 8B). A slight increase was recorded in AP height, NaT, K(DR), NaP, and CaL (Figures 8B2–B6), but only the increase in NaP (Figures 8E3, −0.21 vs. −0.33 nA) and CaL (Figures 8E4, −0.09 vs. −0.14 nA) were shown to be substantial (Figures 8B5,B6, red traces in right panels).

We investigated the effect of channel flicker (CF) on tonic discharges without white noise. Adding CF to NaT generated more spikes compared with control (Figure 8A2 vs. C2), and a slight increase was recorded in NaT currents (Figures 8C3,E1, −5.4 vs. −5.6 nA). Similarly, introducing CF into K(DR), NaP, and CaL also elicited increased repetitive firing (Figures 8C4–C9). The simulation results showed that CF significantly increased NaP (Figures 8E3, −0.21 vs. −0.3 nA) and CaL currents (Figures 8E4, −0.09 vs. −0.2 nA) but not K(DR) currents (Figures 8E2, 1.94 vs. 1.9 nA).

We subsequently examined the effect of CF on the tonic discharge with white noise. We first added CF to NaT, and observed an obvious increase in the number of spikes discharged (Figures 8D2,D3). White noise significantly increased NaT + CF by 0.6 compared with NaT recorded in control (Figure 8E1, *p* < 0.05, *n* = 12 spikes). Similarly, we performed simulations with CF added to K(DR), NaP, and CaL respectively (Figures 8D4–D9), and the simulation results showed that white noise again increased repetitive discharge (Figures 8D4–D9), as well as the inward currents mediated by NaP + CF (Figure 8E3, *p* < 0.05, *n* = 12) and CaL + CF (Figure 8E4, *p* < 0.05, *n* = 12) respectively, compared with NaP and CaL recorded in control. In contrast, however, white noise generated irregular firing with K(DR) + CF (Figure 8D4), and no obvious change was obtained in K(DR) + CF, compared with K(DR) in control, Control + CF, and WN conditions (Figure 8E2).

The results outlined above suggest that white-noise-induced membrane potential oscillations increased NaT, NaP, and CaL and thereby increased repetitive discharge. Moreover, the addition of channel flicker in our models further enhanced the inward currents, especially NaP and CaL which substantially increased the excitability of HG motoneurons.

### 3.8 Channel mechanisms underlying the effects of membrane potential oscillations on spike initiation

**FIGURE 9:**
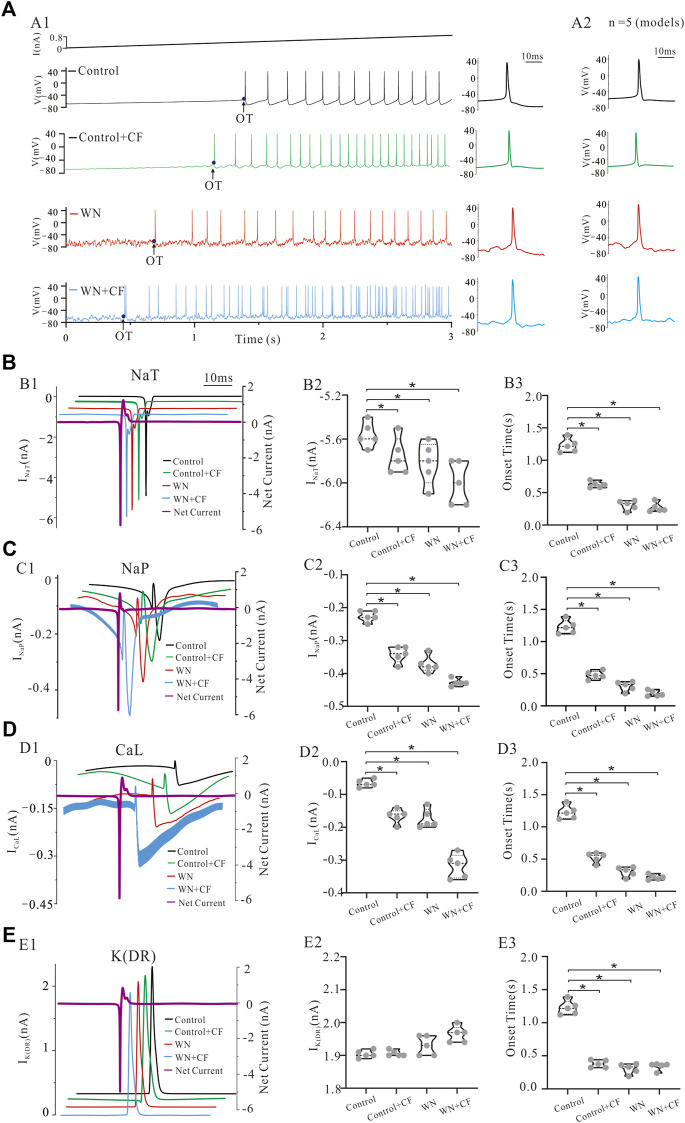
Channel mechanisms underlying membrane potential oscillations effects on spike initiation. The statistical data presented below were recorded with current ramp peak of 1 nA. **(A)** A ramp current with a duration of 3 s and peak of 1 nA was injected into a model HG motoneuron in conditions of control, control + CF, WN and WN + CF, respectively. The onset time (OT) of the first spike of train was recorded, and the averaged APs from **A1** were shown in the right insets (control: black; Control + CF: green; WN: red; WN + CF: blue), and the averaged APs calculated from the five modeled HG motorneurons (Table 1) with the same conditions were shown in **A2**. **(B)** Ionic currents recorded in control, Control + CF, WN and WN + CF. Control + CF (NaT), WN, and WN + CF (NaT) slightly increased NaT currents **(B1,B2)** thus increasing the net inward current (B1, purple) and dramatically shortened the OT of repetitive firing **(B3)**. **(C)** Control + CF (NaP), WN, and WN + CF (NaP) also significantly increased NaP currents **(C1, C2)** thus increasing the net inward current (**C1**, purple) and substantially reduced the OT of the firing train **(C3)**. The increased amount of NaP currents recorded in Control + CF, WN and WN + CF was much larger than that of NaP currents in control. **(D)** Control + CF (CaL), WN, and WN + CF (CaL) substantially increased CaL currents **(D1, D2)** thus increasing the net inward current (D1, purple) and largely shortened the OT of repetitive firing **(D3)**. **(E)** Control + CF (K(DR)), WN, and WN + CF (K(DR)) induced tittle change in K(DR) currents **(E1, E2)** thus maintaining net inward current unchanged (E1, purple). However, K(DR)+CF shortened the OT of repetitive firing with or without WN **(E3)**. Data are expressed as mean ± SD. Unpaired T-tests was performed. *p < 0.05.

It is generally known that the membrane channels underlying the NaT and K(DR) currents are mainly responsible for the generation of single action potentials in motoneurons and that channels mediating other currents such as NaP and CaL facilitate repetitive firing (rev in Powers and Binder, 2001). However, little is known about the roles of the channels mediating these currents in regulating neuronal excitability and initiating spikes in the presence of membrane potential oscillations. To address this issue, we simulated the effects of injected current ramps into five different HG motoneuron models (Figure 8; Table 1) and then averaged the first 12 spikes generated for models corresponding to NaT, NaP, CaL, and K(DR) in control, Control + CF (CF), white noise (WN), and white noise with channel flicker (WN + CF), respectively (Figure 9A1). We calculated the mean AP of the five averaged APs (Figure 9A2) and analyzed the averaged ionic currents underlying the mean AP (Figures 9B–E). Simulation results showed that white noise significantly increased NaT for the AP generation with or without CF. Introducing CF to NaT also significantly increased NaT without white noise (Figure 9B1), leading to an increase in net inward current (Figure 9B1, purple curve). However, the increased amount of NaT was small (<0.5 nA, *p* < 0.05, Figure 9B2). There was no significant difference in the increment between NaT and NaT + CF, suggesting that NaT might play the same role in initiating spikes during membrane potential oscillations regardless of the CF was added to NaT. On the other hand, however, CF and white noise significantly shortened the onset time (OT) for the first spike of the train (Figure 9B3). Moreover, the onset time for WN + CF (NaT) was much shorter than that for Control + CF (Figure 9B3), suggesting that both WN and CF contributed to the generation of action potentials. The differences between NaT and NaP with or without the CF could be largely increased by white noise (Figure 9C1, green), and an increase in NaP could be obtained by simply adding CF to NaP without WN (Figure 9C2). In general, the increased amount of NaP was relatively large and significant increments were observed in the conditions of Control + CF, WN, and WN + CF, indicating that membrane potential oscillations generate large fluctuations in net inward current (Figure 9C1, purple curve) and amplify the functional role of NaP in initiating spikes (Figure 9C2). Control + CF, WN, and WN + CF largely shorten the onset time for generation of the first spike of the train (Figure 9C3), and the shortest onset time was dramatically induced by WN + CF (Figure 9C3).

Similar to NaP, CaL appears to play an essential role in regulating neuronal excitability in the presence of membrane potential oscillations. Adding CF, significantly increased CaL without WN (Figures 9D1,D2). Furthermore, white noise substantially increased CaL with or without CF (Figures 9D1,D2). Statistical analysis of our results showed increases in the calcium current contributions to the net inward current in Control + CF, WN, and WN + CF conditions (Figure 9D2, *p* < 0.05; Figure 9D1, purple curve). The onset time for the first spike was largely reduced by Control + CF, WN, and WN + CF, and the reduction by WN + CF was much larger than that by CF or WN (Figure 9D3).

Finally, we studied the response of K(DR) to white-noise-mediated membrane potential oscillations. The simulation results indicated that K(DR) was not substantially changed by white noise and/or the addition of the CF (Figure 9E1), and no significant alteration of K(DR) was induced by Conotrol + CF, WN, and WN + CF (Figure 9E2). Since K(DR) is an outward current, the net inward current was not reduced by K(DR) significantly (Figure 9E1, purple curve). Interestingly, the onset time for the first spike of train was largely reduced by K(DR) with or without white noise (Figure 9E3), suggesting a role of K(DR)+CF in initiating spikes. Comparing these simulation results with those obtained by manipulating NaT, NaP, and CaL, suggest that K(DR) might play a more significant role in generating action potentials than in regulating the overall excitability of HG motoneurons during white-noise-induced membrane potential oscillations.

To summarize, our simulations demonstrate that the different types of membrane channels involved in regulating motoneuronal excitability and repetitive discharge respond differently to noise induced membrane potential oscillations. Among the four channels, NaP and CaL appear to contribute more to the regulation of the excitability total output of HG motoneurons in the presence of membrane potential oscillations, whereas NaT and K(DR) appear to contribute more to the variability of repetitive discharge patterns under the same conditions.

## 4 Discussion

Using a combination of electrophysiological and modeling approaches, we have investigated the effect of white-noise-induced membrane potential oscillations on the excitability of hypoglossal motoneurons in rats. Our experimental data showed that white noise increased the excitability of HG motoneurons manifested by a reduction of the recruitment current for repetitive firing, hyperpolarization of voltage threshold for the first spike initiation, and prolongation of the repetitive firing. Our HG motoneuron models based on conventional Hodgkin-Huxley formulations effectively replicated our experimental results except for the F-I relationships. To reproduce the F-I curves more closely, we embellished our models with a novel channel flicker (CF) term corresponding to the noise generated by the stochastic gating behavior of voltage-gated ion channels (White et al., 2000; Dorval and White, 2005; Dorval, 2006). Simulation results indicated that adding the CF modulation to the channels largely increased the neuronal output and excitability, closely mimicked the experimental F-I curves, and significantly prolonged repetitive discharges evoked by current ramps as we had observed experimentally. Further analysis suggested that the channel flicker increased the net inward currents for initiation of spikes and regulation of neuronal excitability.

### 4.1 Membrane properties changed with slope of current ramps

In this study, we specifically explored the noise-induced changes in membrane properties with respect to the slopes of current ramp. It has been reported that spinal motoneurons are more readily activated by injected currents as their rate of rise increases (Dai et al., 2000; Dai and Jordan, 2011). However, both the experimental and modeling results reported here for rat brainstem hypoglossal motoneurons did not support this prior finding with respect to the changes in V_th_ (Figure 2). In fact, V_th_ did not significantly change with increase of ramp slopes under both control and noise conditions in both our experimental (Figures 2A,B) and modeling results (Figures 2C,D). In contrast, I_rec_ and ΔI (=I_dec_-I_rec_) appeared to increase with increment of the ramp slopes (peaks) in the rat experiments (Figures 3A,B), and this was mimicked by modeling (Figure 3C,D). These results suggested that the slopes of current ramps used in the present study might not be steep or fast enough to activate stronger sodium or calcium currents to alter the spike initiation at subthreshold potentials, but substantially strong enough to enhance the interactions between NaT and K(DR) for repetitive firing. It is of course also possible that the activation of HG motoneurons in response to currents injected into the soma differ from those in spinal motoneurons. It was these discrepancies that prompted us to introduce channel flicker (CF) into the kinetics of the membrane channels in our models.

### 4.2 Membrane properties changed with channel flicker

We found that adding a CF significantly altered the model HG motoneuron’s responses to noise-induced membrane potential oscillations. Our models with CF effectively reproduced our major experimental results including noise-induced decrease in V_th_, I_rec_, and ΔI. Hyperpolarization of V_th_ was observed only in the first spike of repetitive firing (Figures 2A,B), similar to the results obtained in our prior study of cat lumbar motoneurons (Dai et al., 2000). This phenomenon was due to fast activation of NaT channels rather than neuromodulation of the channels. However, there were differences between the experimental results and modeling simulations with respect to the F-I relationships. The present experimental data showed that there was no substantial change in the slope and intercept of the F-I curves between control and noise experiments (Figures 4D–F, intercept: *p* = 0.23, slope: *p* = 0.08), whereas a significant increase in slope and left-shifting of the F-I curves appeared in the simulation results (Figure 4G, intercept: *p* = 0.007, slope: *p* = 0.001) collected from averaged data from five HG motoneuron models (Table 1). However, an overall increase in HG motoneuron firing rate had been reported previously by the Binder lab (Poliakov et al., 1996). In attempt to reconcile the differences between the F-I curves and the slope-related activation properties between cat spinal and rat HG motoneurons, we introduced a channel flicker (CF) modification to HH-type channel kinetic equations. A similar mechanism termed stochastic flicker (SF) was described previously in studies of channel noise for perithreshold oscillations in entorhinal stellate neurons (Dorval and White 2005; Dorval, 2006). The channels appear to “flicker” between open and closed states, and thus the rate constants were expressed in those studies as a function of membrane potential and combined to determine the average open probability and standard deviation of channel states. The CF used in the present study, however, is different from the SF in Dorval et al. studies. Here, the CF modulates channel conductance to increase the motoneurorons’ sensitivity to membrane potential oscillations, whereas the SF is a major mechanism underlying the slow perithreshold oscillations. To our knowledge, this is the first time that sine functions have been added to standard HH-type equations to mimic channel modulation. Our rationale for constructing our CF as we did was that we thought it would be the simplest way to capture the striking periodicity in the membrane potential oscillations observed in spinal neurons recorded in the presence of 5-HT (Dai et al., 2009) and during fictive locomotion (MacDonell et al., 2015). In the present study, we simplified the representation of CF as a sinusoidal function which directly affected the conductance of channels rather than their gating kinetics. The CF is determined by the amplitude (a) and frequency (b) of the sin function. The values of a and b were adjusted so that the channel-mediated currents are substantially increased while the membrane properties of the modeled HG motoneurons remain unchanged (Table 1). In the present model, the values of (a, b) in NaT, K(DR), NaP, and CaL are set to (0.1, 0.1), (0.1, 0.1), (0.04, 0.1), and (0.03, 0.1), respectively. The channel flicker amplified the channel sensitivity to the noise-induced membrane oscillations and generated the F-I curves that closely resembled those recorded experimentally. The simulation results showed that the WN + CF (NaP) and WN + CF (CaL) conditions with CF produced F-I curves (Figures 5E,F, green lines) more consistent with the experimental ones (Figures 5E,F, red line) than NaP and CaL without CF (Figures 5E,F, black and blue lines). Also, WN + CF (NaP) and WN + CF (CaL) generated F-I curves more comparable to experimental data than NaT and K(DR) with or without CF (Figures 5G,H). On the other hand, the extent of ΔI reduction induced by WN + CF (NaP) and WN + CF (CaL) (Figures 7D–F) was larger than that induced by WN + CF (NaT) and WN + CF [K(DR)], respectively (Figures 7B–H). Analysis of membrane potential oscillation-induced inward currents also indicated that WN + CF (NaP) and WN + CF (CaL) generated much larger inward currents than NaT and K(DR) with or without WN (Figures 8E, 9B–E) thus indicating that NaP and CaL to play more important roles in regulating neuronal excitability than NaT and K(DR) in response to noise-induced membrane potential oscillations.

Finally, we should emphasize that the channel flicker (CF) term used in the present models is distinct from the stochastic flicker (SF) described by Dorval and White, (2005), which was derived from the stochastic channel openings and closings that they observed in their experimental study of NaP (Dorval and White, 2005; Dorval, 2006). Unlike Dorval’s work on stellate neurons, we have no experimental evidence to confirm the existence of the CF in rat HG motoneurons: it is simply an imputed variable that was added to the standard Hodgkin-Huxley formulation to provide a better match to the experimental measurements of F-I relationships and discharge duration that we made in rat hypoglossal motoneurons. There are three differences between the CF used in the present models and the SF in Dorval’s work: 1) The CF modulates channel conductance. Whereas the SF regulates the gating property (state variables of activation); 2) The CF is added to the channel conductance to increase the neuronal sensitivity to the membrane potential oscillation, whereas the SF is a key mechanism responsible for the slow perithreshold oscillations in stellate neurons of the entorhinal cortex. And 3) The CF is described by a sine wave function whereas the SF is determined by the state variable (activation) of NaP with an adjustable standard deviation. Again, though we have no experimental evidence to confirm the existence of the CF in rat HG motoneurons, introducing CF into our models greatly enhanced the capacity of our models to reproduce the changes in the F-I relationships and the prolonged discharges observed in real HG motoneurons during white noise-induced membrane potential oscillation. Furthermore, adding CF to ion channels does not alter the membrane properties of HG motoneurons in Table 1. Additional experiments are required to verify the predictions of the model.

### 4.3 Mechanisms regulating excitability of hypoglossal motoneurons with channel flicker

Our simulation results indicated that channel flicker (CF) clearly enhanced motoneuron responses to the membrane potential oscillations, particularly the F-I relationships. Motoneuron firing properties are determined not only by synaptic input but also by the behavior of voltage-gated channels including NaT (Dai et al., 2002; Power et al., 2012), K(DR) (Lombardo and Harrington, 2016; Dai et al., 2018), CaL (Schwindt and Crill, 1980a; Schwindt and Crill, 1980b; Heckman et al., 2003; Powers et al., 2012; Binder et al., 2020), and NaP (Crill, 1996; Lee and Heckman, 2001; Zhong et al., 2006; Brocard, 2019). In this study, we further explored the channel mechanisms underlying changes in the excitability of HG motoneurons induced by membrane potential oscillations. The simulations revealed that the membrane noise induced larger amplitudes of NaT, K(DR), NaP, and CaL currents with CF in place than those without CF, thus altering the threshold for generation of action potentials (Figure 8). Further analysis indicated that the CF amplified the net inward current on rising phase of the spikes (Figures 9B1–E1) and shortened the onset time for spike initiation (Figures 9B3–E3). CF added to different channel types induced varying effects on inward currents, suggesting different roles for specific channels in regulating the neuronal output and excitability. While adding CF increased NaT currents (Figures 9B1,B2) and facilitated net inward currents for the action potential generation, the K(DR) currents remained almost unchanged (Figures 9E1,E2), thus indirectly increasing the net inward current by reducing the outward currents. Large inward currents were generated by NaP (Figures 9C1,C2) and CaL channels (Figures 9D1,D2), and more importantly the net inward current on rising phase of the action potential was largely increased by NaP and CaL with CF, giving rise to a higher probability of spike initiation and repetitive firing in noise-induced membrane oscillation. In summary, CF altered the channel kinetics and enhanced the HG motoneuron response to white noise-induced membrane oscillations, without changing the basic gating property of the channels. Thus, it appears that the CF of channels responsible for NaP and CaL play a major role in regulating the excitability and output of HG motoneurons, while the CF in those channels underlying NaT and K(DR) appear to be mainly responsible for triggering the large variability of repetitive firing in HG motoneurons.

### 4.4 Construction of hypoglossal motoneuron models

Through the review process, we became aware of a similar HG motoneuron model constructed with nine types of channels (Purvis and Butera 2005). However, any model is an approximation and, in our opinion, the complexity of a model should depend on the specific issues under examination. Here, we have constructed the models to focus exclusively on the alteration of excitability induced by membrane potential oscillations. At the outset, we established seven principal membrane properties of real HG motoneurons, those essential for regulating neuronal excitability, as the “target values” for our models (Table 1). With such constraints imposed on the membrane properties, we were delighted that our models provided such impressive fits to the experimental results we collected. Nonetheless, the Purvis and Buteral model (2005) might offer some improvement and we will consider adopting it in our future studies.

## 5 Conclusion

We found that white membrane noise altered the excitability of rat HG motoneurons. The noise-induced changes in membrane properties could be adequately fit by HG motoneuron models. However, adding a channel flicker (CF) term to the behavior of the membrane channels in our models was required to enhance the inward currents sufficiently to reproduce the F-I relationships and prolongation of repetitive firing observed in real rat HG motoneurons with white noise-induced membrane potential oscillations. With CF included in the models, it appears that the channels underlying NaP and CaL were mainly responsible for the regulation of neuronal excitability and output, whereas the channels mediating NaT and K(DR) appear to be largely responsible for the regularity of repetitive firing in HG motoneurons.

## Data Availability

The raw data supporting the conclusion of this article will be made available by the authors, without undue reservation.
