# Supplementary material for: The effects of membrane potential oscillations on the excitability of rat hypoglossal motoneurons
Source: Front Physiol. 2022 Aug 23;13:955566. doi: 10.3389/fphys.2022.955566 (PMC9445839; doi:10.3389/fphys.2022.955566)
Supplement: Supplementary file 1 [file DataSheet1.docx]

**Supplementary Appendix**

State variables *m*, *h*, *n*, and *s* were defined by Hodgkin-Huxley equation as below:

| $\frac{dX}{dt}=\alpha(1-X)-\beta X$ | (1) |
| --- | --- |

where steady-state value $X_{\infty}=\alpha/(\alpha+\beta)$and time constant$\tau=1/(\alpha+\beta)$.

NaT channel：

The $m_{\infty}$and $\tau_{m}$ was described by the following equations:

| $m_{\infty}=\frac{\frac{0.4\cdot(V_{m}+29)}{1-\exp[-(V_{m}+29)/7.2]}}{\frac{0.4\cdot(V_{m}+29)}{1-\exp[-(V_{m}+29)/7.2]}+\frac{0.124\cdot(V_{m}+29)}{1-\exp[(V_{m}+29)/7.2]}}$ | (2) |
| --- | --- |
| $\tau_{m}=\frac{1}{\frac{0.4\cdot(V_{m}+29)}{1-\exp[-(V_{m}+29)/7.2]}+\frac{0.124\cdot(V_{m}+29)}{1-\exp[(V_{m}+29)/7.2]}}$ | (3) |

The $h_{\infty}$and $\tau_{h}$ was described by the following equations:

| $h_{\infty}=\frac{\frac{0.06\cdot(V_{m}+49)}{1-\exp[-(V_{m}+49)/1.3]}}{\frac{0.06\cdot(V_{m}+49)}{1-\exp[-(V_{m}+49)/1.3]}+\frac{0.01\cdot(V_{m}+49)}{1-\exp[(V_{m}+49)/1.3]}}$ | (4) |
| --- | --- |
| $\tau_{h}=\frac{1}{\frac{0.06\cdot(V_{m}+44)}{1-\exp[-(V_{m}+44)/1.3]}+\frac{0.01\cdot(V_{m}+44)}{1-\exp[(V_{m}+44)/1.3]}}$ | (5) |

NaP channel:

The $m_{\infty}$and $\tau_{m}$ was described by the following equations:

| $m_{\infty}=\frac{1}{1+\exp[-(V_{m}+47.3)/5]}$ | (6) |
| --- | --- |
| $\tau_{m}=1$ | (7) |

The $s_{\infty}$and $\tau_{s}$ was described by the following equations:

| $s_{\infty}=\frac{0.001\cdot\exp[-(V_{m}+90)/30]}{0.001\cdot\exp[-(V_{m}+90)/30]+0.034\cdot\exp[-(V_{m}+22)/10]}$ | (8) |
| --- | --- |
| $\tau_{s}=\frac{1}{0.001\cdot\exp[-(V_{m}+90)/30]+0.034\cdot\exp[-(V_{m}+22)/10]}$ | (9) |

K(DR) channel:

The $n_{\infty}$and $\tau_{n}$ was described by the following equations:

| $n_{\infty}=\frac{1}{1+\exp[-(V_{m}+21)/20]}$ | (10) |
| --- | --- |
| $\tau_{n}=0.8\text{+}\frac{20\cdot\exp[(V_{m}+39)/5.5]}{\{1+\exp[(V_{m}+39)/5.5]\}^{2}}$ | (11) |

K(AHP) channel:

The $q_{\infty}$and $\tau_{q}$ was described by the following equations:

| $q_{\infty}=\frac{0.1\{[Ca^{2+}]_{in}\text{-}[Ca^{2+}]_{\infty}\}^{2}}{0.1\{[Ca^{2+}]_{in}\text{-}[Ca^{2+}]_{\infty}\}^{2}+0.1}$ | (12) |
| --- | --- |
| $\tau_{q}=\frac{1}{0.1\{[Ca^{2+}]_{in}\text{-}[Ca^{2+}]_{\infty}\}^{2}+0.1}$ | (13) |

where [Ca^2+^]_in_ was the intracellular calcium concentration. The equations governing the Ca^2+^-dependent potassium channels (K(AHP)) were similar to those used by Sah (1992) and the mathematical equations are as follows：

| $\frac{d[Ca^{2+}]_{in}}{dt}=\frac{[Ca^{2+}]_{\infty}-[Ca^{2+}]_{in}}{\tau_{Ca}}-\frac{i_{Ca}}{2\cdot F\cdot d}$ | (14) |
| --- | --- |

where *τ_Ca_* is the time constant of decay and the value of *τ_Ca_* is 60 ms, *i_Ca_* is Ca^2+^ current density, *F* is Faraday’s constant, and *d* is the depth of the inner shell around the K(AHP) channels and the value of *d* is 0.1μm.

H channel:

The $m_{\infty}$and $\tau_{\text{m}}$ was described by the following equations:

| $m_{\infty}=1/1+\exp[(V_{m}+77)/8]$ | (15) |
| --- | --- |
| $\tau_{m}=40$ | (16) |

CaL channel:

The $m_{\infty}$and $\tau_{m}$ was described by the following equations:

| $m_{\infty}=1/1+\exp[(V_{m}+40)/6]$ | (17) |
| --- | --- |
| $\tau_{m}=40$ | (18) |
